# Nonhuman primates across sub-Saharan Africa are infected with the yaws bacterium *Treponema pallidum* subsp. *pertenue*

**DOI:** 10.1038/s41426-018-0156-4

**Published:** 2018-09-19

**Authors:** Sascha Knauf, Jan F. Gogarten, Verena J. Schuenemann, Hélène M. De Nys, Ariane Düx, Michal Strouhal, Lenka Mikalová, Kirsten I. Bos, Roy Armstrong, Emmanuel K. Batamuzi, Idrissa S. Chuma, Bernard Davoust, Georges Diatta, Robert D. Fyumagwa, Reuben R. Kazwala, Julius D. Keyyu, Inyasi A. V. Lejora, Anthony Levasseur, Hsi Liu, Michael A. Mayhew, Oleg Mediannikov, Didier Raoult, Roman M. Wittig, Christian Roos, Fabian H. Leendertz, David Šmajs, Kay Nieselt, Johannes Krause, Sébastien Calvignac-Spencer

**Affiliations:** 1 Work Group Neglected Tropical Diseases, Infection Biology Unit, German Primate Center, Leibniz Institute for Primate Research, Kellnerweg 4, 37077 Göttingen, Germany; 2 Epidemiology of Highly Pathogenic Microorganisms, Robert Koch Institute, Seestraße 10, 13353 Berlin, Germany; 3 Primatology Department, Max Planck Institute for Evolutionary Anthropology, Deutscher Pl. 6, 04103 Leipzig, Germany; 4 Department of Biology, McGill University, 1205 Avenue du Docteur-Penfield, Montréal, QC H3A 1B1 Quebec Canada; 5 Institute for Archaeological Sciences, University of Tübingen, Ruemelinstrasse 23, 72070 Tübingen, Germany; 6 Viral Evolution, Robert Koch Institute, Seestraße 10, 13353 Berlin, Germany; 7 Department of Biology, Faculty of Medicine, Masaryk University, Kamenice 5/A6, 625 00 Brno, Czech Republic; 8 Max Planck Institute for the Science of Human History, Kahlaische Strasse 10, 7745 Jena, Germany; 9 Department of Science, Natural Resources and Outdoor Studies, University of Cumbria, Fusehill Street, Carlisle, Cumbria CA12HH UK; 10 Department of Veterinary Surgery and Theriogenology, College of Veterinary and Medical Sciences, Sokoine University of Agriculture, Morogoro, Tanzania; 11 Ecological Monitoring Department, Tanzania National Parks (TANAPA), P.O.Box 3134 Arusha, Tanzania; 12 Research Unit of Emerging Infectious and Tropical Diseases (URMITE) UM63, CNRS 7278, IRD 198, INSERM 1095, Aix-Marseille University, 27, Bd Jean Moulin, 13385 Marseille cedex 05, Marseille, France; 13 Research Unit of Emerging Infectious and Tropical Diseases (URMITE) IRD 198, Campus IRD/UCAD, Hann Les Maristes, Dakar, Senegal; 14 Tanzania Wildlife Research Institute (TAWIRI), P.O. Box 661 Arusha, Tanzania; 15 Department of Veterinary Medicine and Public Health, College of Veterinary and Medical Sciences, Sokoine University of Agriculture, Morogoro, Tanzania; 16 National Center for HIV/AIDS, Viral Hepatitis, STD, and TB Prevention, Centers for Diseases Control and Prevention, Atlanta, GA USA; 17 Taï Chimpanzee Project, Centre Suisse de Recherches Scientifiques, BP 1303, Abidjan 01, Abidjan, Côte d’Ivoire; 18 Gene Bank of Primates and Primate Genetics Laboratory, German Primate Center, Leibniz Institute for Primate Research, Kellnerweg 4, 37077 Göttingen, Germany; 19 Center for Bioinformatics, University of Tübingen, Sand 14, 72076 Tübingen, Germany; 20 Institute of Evolutionary Medicine, University of Zurich, Winterthurerstrasse 190, 8057 Zurich, Switzerland; 21 Present Address: Unité Mixte Internationale 233, Institut de Recherche pour le Développement, INSERM U1175, and University of Montpellier, Montpellier, France

Dear Editor,

The bacterium *Treponema pallidum* (*TP*) causes human syphilis (subsp. *pallidum*; *TPA*), bejel (subsp. *endemicum*; *TEN*), and yaws (subsp. *pertenue*; *TPE*)^1^. Although syphilis has reached a worldwide distribution^2^, bejel and yaws have remained endemic diseases. Bejel affects individuals in dry areas of Sahelian Africa and Saudi Arabia, whereas yaws affects those living in the humid tropics^1^. Yaws is currently reported as endemic in 14 countries, and an additional 84 countries have a known history of yaws but lack recent epidemiological data^3,4^. Although this disease was subject to global eradication efforts in the mid-20th century, it later reemerged in West Africa, Southern Asia, and the Pacific region^5^. New large-scale treatment options triggered the ongoing second eradication campaign, the goal of which is to eradicate yaws globally by 2020^5^.

*TPE* is typically considered to be a strictly human pathogen, a perception that may partially have arisen from a lack of detailed data on nonhuman primate (NHP)-infecting treponemes. Indeed, a number of African NHPs show skin ulcerations that are suggestive of treponemal infections, and antibodies against *TP* have been detected in wild NHP populations^6,7^. Although genetic studies confirmed that monkeys and great apes are infected with *TP* strains^8–10^, most of these analyses only used short DNA sequences. Thus, the small number of examined polymorphic sites largely precluded assignment of these strains to a particular *TP* subspecies^9^, especially considering that sporadic recombination events between subspecies have been reported^11^. The only simian strain whose whole genome has been sequenced (Fribourg-Blanc, isolated from a Guinea baboon (*Papio papio*) in 1966^7^) unambiguously clustered with human-infecting *TPE* strains^12^.

A fundamental question with regard to yaws evolution, and possibly yaws eradication, is whether humans and NHPs are commonly infected with the same pathogen (*TPE*) and whether transmission between NHPs and humans occurs. To determine which pathogen causes treponematoses in NHPs across sub-Saharan Africa, we collected samples from symptomatic wild individuals belonging to three NHP species (*Cercocebus atys*, *Chlorocebus sabaeus*, and *Papio anubis*) from four independent populations in West and East Africa (Fig. 1, Supplementary Table S1, Supplementary Materials). Samples were collected from NHPs at Taï National Park (TaïNP; Côte d’Ivoire), Bijilo Forest Park (BFP, the Gambia), Niokolo-Koba National Park (NKNP, Senegal), and Lake Manyara National Park (LMNP, Tanzania). Monkeys presented yaws-like orofacial and limb lesions (TaïNP and BFP) or ulcerative anogenital skin lesions (BFP, NKNP, and LMNP)^9^.

**Fig. 1:**
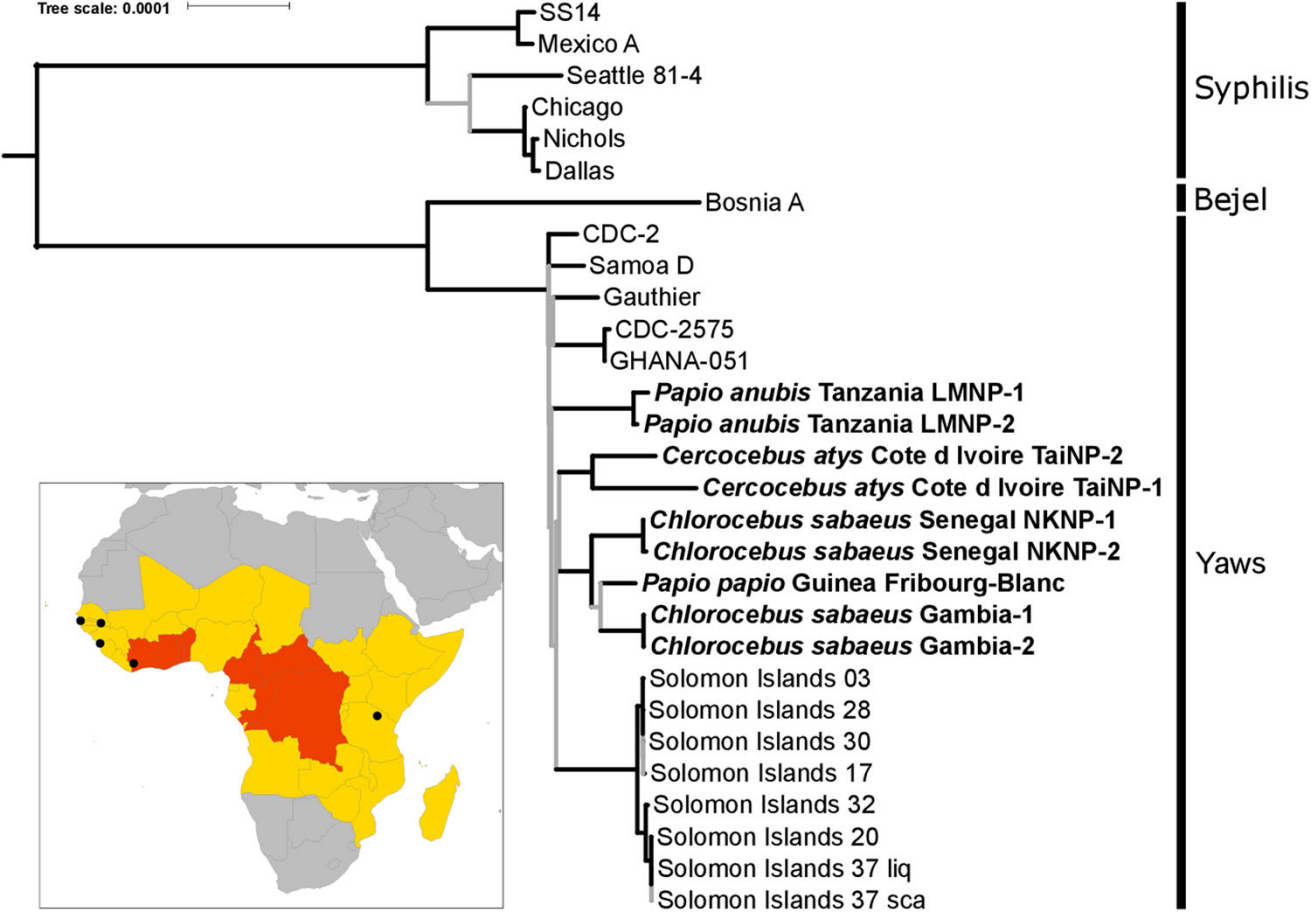
Phylogenomic analysis of NHP- and human-infecting *Treponema pallidum* strains. NHP-infecting *Treponema pallidum* strains are indicated in bold. In this maximum likelihood tree, nodes with less than 95% bootstrap approximation support are indicated with gray lines. Tip labels indicate the NHP species sampled, the country of origin, and the sample ID. The scale is in nucleotide substitutions per site. The inset is a map of Africa showing the sites of origin of NHP samples from which a *TP* genome was determined (indicated with black circles). The 2013 yaws status of countries, based on the World Health Organization’s Global Health Observatory (http://www.who.int/gho/en/), are indicated by color: gray indicates no previous history of yaws infections in humans, yellow indicates a country previously endemic for yaws for which the current status is unknown, and red indicates countries that are currently considered endemic for yaws

Using a PCR-based assay, we demonstrated the presence of *TP* in skin lesion biopsies or swabs from NHPs inhabiting TaïNP (*C. atys*), BFP, and NKNP (*C. sabaeus*). *TP* infection in olive baboons (*P. anubis*) from LMNP had previously been confirmed^6^. Two samples per NHP population were selected for whole-genome sequencing based on a high *TP* copy number or the ability to amplify long PCR fragments (Supplementary Table S2). To overcome the presence of background host genomic DNA, we used targeted DNA capture coupled with next generation sequencing to reconstruct whole *TP* genomes^2,8^. Following quality filtering, removal of PCR duplicates, merging of different sequencing runs from the same sample, and mapping against the *TPE* strain Fribourg-Blanc reference genome, we obtained a range of 22,886–470,303 DNA sequencing reads per sample. All samples showed at least an 80% coverage of the reference genome with a depth coverage of three or higher; the average genome coverage depth was between 6.1-fold and 121.0-fold (Supplementary Table S3).

We generated maximum likelihood, Bayesian and maximum parsimony trees based on the genomes reconstructed in our study and all available reference genomes (total sequence length: 1,133,379 nucleotides). In all trees, the *TPE* and *TPA* strains formed reciprocally monophyletic groups, with a mean *TPE*/*TPA* strain divergence of 0.099%. NHP-infecting *TP* strains all clustered with human-infecting *TPE* strains (Fig. 1; Supplementary Figure S1). The *TPE* clade exhibited a star-like branching pattern with basal branches that were very short and received low statistical support. Importantly, this pattern does not support a clear reciprocal monophyly of the *TPE* strains infecting humans and NHPs. In line with this result, the minimum divergence between strains infecting humans and NHPs was lower than the maximum divergence among human or NHP-infecting strains (0.011% versus 0.015% and 0.024%). The human-infecting *TPE* strains Samoa D, CDC-2, CDC-2575, Ghana-051, and Gauthier, which span a broad geographic and temporal range (at least four decades), were less divergent from each other than the two strains infecting sooty mangabeys from a single social group at TaïNP, which were collected in the same week (0.011% versus 0.017% sequence divergence, respectively). While intra-group strain divergence was low for the two African green monkey populations and the olive baboons (0.0003% and 0.0017%, respectively), intra-species strain divergence among African green monkeys was relatively high compared to the divergence observed between the two most divergent human strains (0.0094% versus 0.015%).

We determined the complete genome sequence and structure for the *TPE* strain from sample LMNP-1 (average depth of coverage: ×169; GenBank: CP021113; Supplementary Table S5-6)^12^. The genome structure of the LMNP-1 strain was the same as those of published complete genomes of human-infecting *TPE* strains and that of the simian strain Fribourg-Blanc. Furthermore, the genome of the LMNP-1 strain was more similar to that of the human-infecting *TPE* Gauthier strain than the simian isolate Fribourg-Blanc, showing differences at 266 and 325 chromosomal positions, respectively. Most differences were single-nucleotide substitutions or small indels (Supplementary Table S7). The LMNP-1 and Gauthier strains exhibited the same number of 24-bp repeats in the *TP_0470* gene (*n* = 25), and the Gauthier strain had only one 60-bp repeat more than the LMNP-1 strain in the *arp* gene (LMNP-1 *n* = 9 vs. Gauthier *n* = 10). All 60-bp repeats in the *arp* gene of the LMNP-1 strain were of Type II and were identical to other *TPE* strains^13^. The *tprK* gene of the LMNP-1 strain had only three variable regions, V5–V7, compared to other *TPE* strains. In addition to differences in the *TP_0433*, *TP_0470*, and *tprK* genes, relatively large indels were identified in *TPEGAU_0136* (33-nt long deletion; specific for the strains Gauthier and Samoa D), *TPFB_0548* (42-nt long deletion; specific for strain Fribourg-Blanc), and *TPEGAU_0858* (79-nt long deletion; specific for strain Gauthier), and in the intergenic regions (IGRs) between *TPEGAU_0628* and *TPEGAU_0629* (302-nt long deletion; specific for strain Gauthier) and *TPFB_0696* and *TPFB_0697* (430-nt long insertion; specific for strain Fribourg-Blanc); the lengths of the other sequence differences ranged between 1 and 15 nt. The structures of the rRNA operons in the LMNP-1 genome (coordinates 231,180–236,139; 279,584–284,533; according to *TPE* strain Gauthier: NC_016843.1) were similar to those in strains Gauthier, CDC-2, and Fribourg-Blanc, but were different than those in strains Samoa D, Samoa F, and CDC-1. The LMNP-1 16S–5S–23S region was identical in both operons, and the 23S rRNA sequences were identical to those in other *TPE* strains except for strain Fribourg-Blanc (having a single-nucleotide difference at position 458). We did not observe any mutations associated with macrolide resistance (e.g., A2058G, A2059G)^14^. When the two NHP-infecting TPE strains (Fribourg-Blanc and LMNP-1) were compared to the closest human-pathogenic TPE strains (CDC-2 and Gauthier) only 7.2 and 9.1% of all coding sequences (77 and 97 coding sequences out of 1065) contained amino acid substitutions, respectively, suggesting limited functional divergence among these strains (Supplementary Table S7-9).

Our findings unambiguously indicate that at least three African NHP species (representing four populations) from West and East Africa currently suffer from treponematosis caused by *TPE*. Taking into account the isolation of the Fribourg-Blanc strain from Guinea baboons in 1966 and its recent sequencing and identification as a member of the *TPE* clade^12^, there are currently four African NHP species and five populations whose symptoms can be explained by *TPE* infections. Coupled with a growing number of clinical and serological observations^6,7,9,10^, these findings suggest that infection of NHPs with *TPE* is common throughout sub-Saharan Africa. Thus, humans are not the exclusive host for the yaws bacterium, as NHPs are infected with the same bacterial agent.

*TPE* strains in NHPs exhibit considerable genetic diversity, which at least equals that found among published human-infecting *TPE* strains. Importantly, we found no evidence for a clear sub-differentiation of NHP-infecting and human-infecting *TPE* strains, i.e., these strains did not form well-supported reciprocally monophyletic groups. Rather, the star-like topology of our phylogenomic tree suggests a rapid initial radiation of the ancestor of *TPE*, which may have involved transmission across primate species barriers in the relatively distant past (with respect to the *TPE* clade depth). These results neither support nor allow us to exclude a possible recent transmission of *TPE* between NHPs and humans, especially due to the large geographic and temporal separation between the two groups of samples compared in this study. A major hurdle in identifying such potential transmission events is the availability of bacterial genomes. Despite large numbers of human cases, very few genomes have been determined from human-infecting *TPE* strains and only from a very limited geographic range. Generating additional human-infecting *TPE* genomes represents an important area of research, the results of which, when coupled with the genomes of the NHP-infecting *TPE* strains presented here, could enable the detection of recent zoonotic transmission events, should any exist.

Since yaws has not been reported for several decades in humans in countries where we observed NHPs to be infected with *TPE*, we expect that if transmission of *TPE* between NHPs and humans occurs, it does so at a very low frequency (as is the case for many zoonotic diseases). Of course, such a low frequency of zoonotic transmission would not alone explain the reemergence of yaws, which is largely (or entirely) the consequence of continued human-to-human transmission. However, now that eradication of yaws appears within reach^15^, the finding that *TPE* strains circulate in NHPs certainly supports the call for more research into their diversity and zoonotic potential.

## Electronic supplementary material

Supplementary Figure S1

Supplementary Table S1

Supplementary Table S2

Supplementary Table S3

Supplementary Table S4

Supplementary Table S5

Supplementary Table S6

Supplementary Table S7

Supplementary Table S8

Supplementary Table S9

Supplementary Materials

## Data Availability

All raw sequence read files have been deposited in NCBI as part of the BioProject PRJNA343706.
