# Supplementary material for: Nonhuman primates across sub-Saharan Africa are infected with the yaws bacterium Treponema pallidum subsp. pertenue
Source: Emerg Microbes Infect. 2018 Sep 19;7:157. doi: 10.1038/s41426-018-0156-4 (PMC6143531; doi:10.1038/s41426-018-0156-4)
Supplement: Supplementary file 1 — Supplementary Figure S1 [file 41426_2018_156_MOESM1_ESM.docx]

**A.**


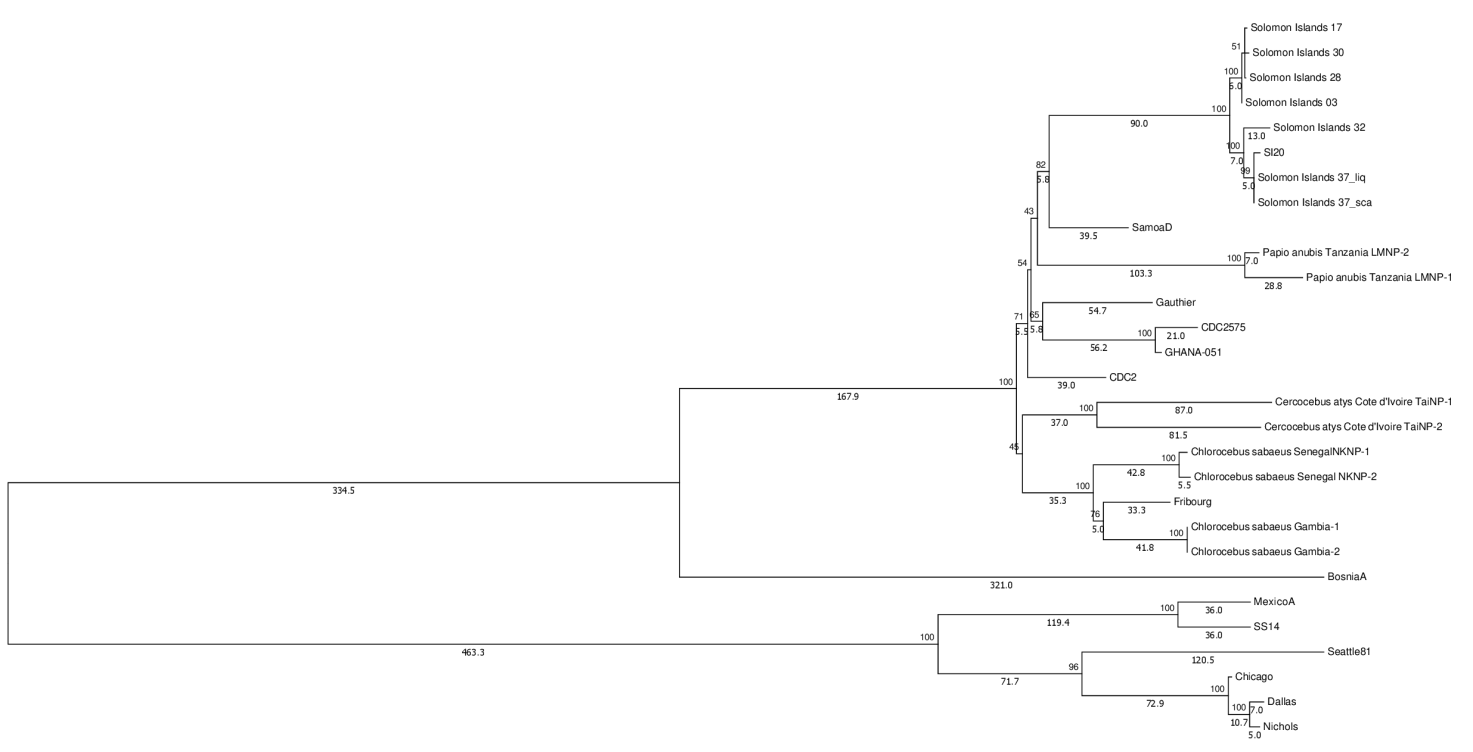


**B.
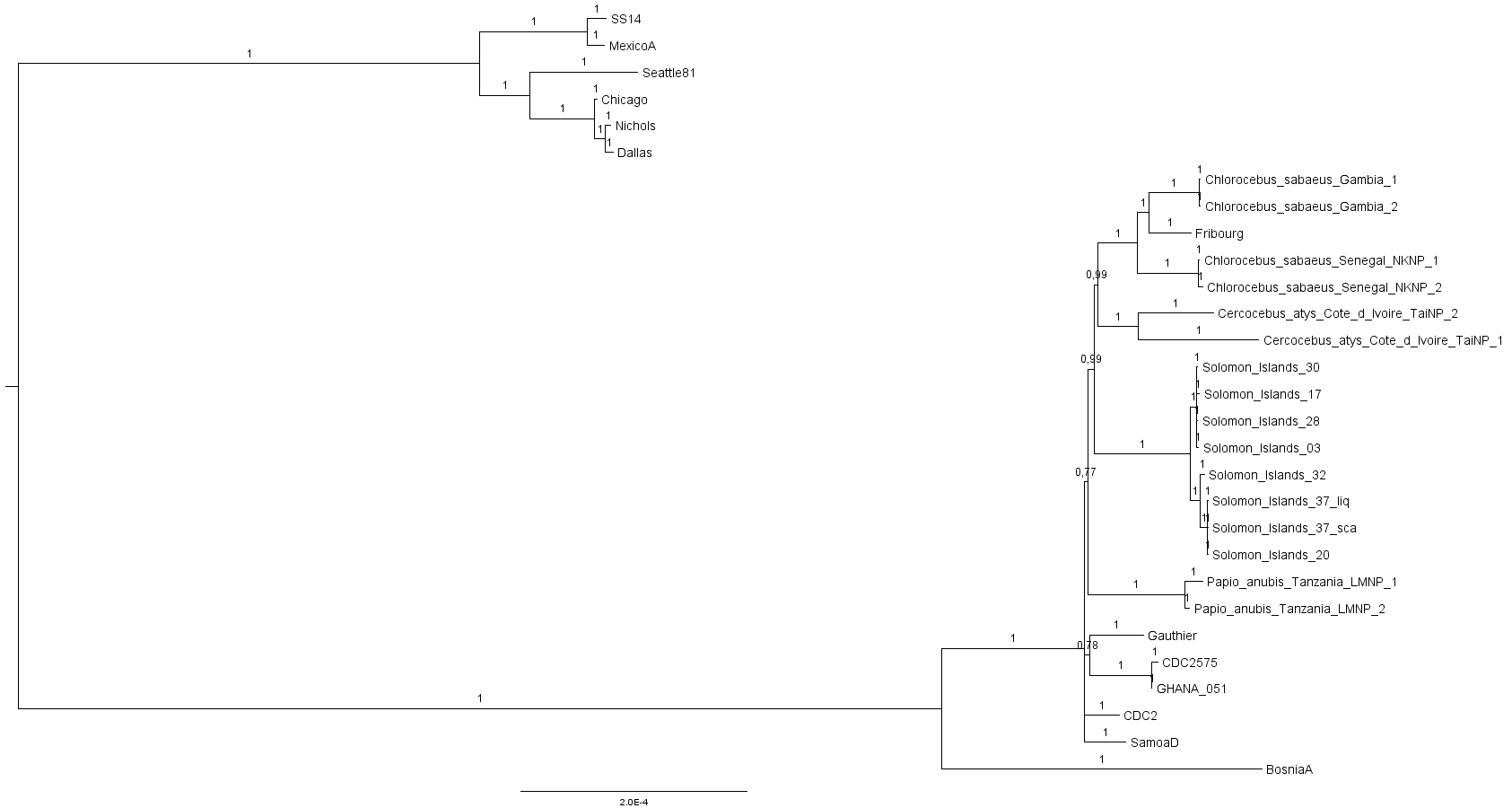
**

**Fig S1.** Phylogenetic trees of *TP* whole genome sequences. A. Maximum parsimony tree. The percentage of replicate trees in which the associated taxa clustered together in the bootstrap test (100 replicates) are shown next to the nodes, above branches. Branch lengths are in the units of the number of changes over the whole sequence and appear below branches. B. Maximum clade credibility tree. Posterior probabilities are plotted above branches. The scale is in substitutions per site across the whole genome.
