# Supplementary material for: Nonhuman primates across sub-Saharan Africa are infected with the yaws bacterium Treponema pallidum subsp. pertenue
Source: Emerg Microbes Infect. 2018 Sep 19;7:157. doi: 10.1038/s41426-018-0156-4 (PMC6143531; doi:10.1038/s41426-018-0156-4)
Supplement: Supplementary file 2 — Supplementary Table S1 [file 41426_2018_156_MOESM2_ESM.docx]

**Table S1.** Nonhuman primates anesthetized for this study.

| Species | Number of individuals | Clinical manifestations | Park, country |
| --- | --- | --- | --- |
| Sooty mangabey  (*Cercocebus atys*) | 5 | Face or lower extremities incl. bone deformation | TaïNP, Côte d’Ivoire |
| African green monkey  (*Chlorocebus sabaeus*) | 5 | Face and anogenital | BFP, The Gambia |
| African green monkey  (*Chlorocebus sabaeus*) | 3 | Genital | NKNP, Senegal |
| Olive baboon  (*Papio anubis*) | 2 | Anogenital | LMNP, Tanzania |
