# Supplementary material for: Nonhuman primates across sub-Saharan Africa are infected with the yaws bacterium Treponema pallidum subsp. pertenue
Source: Emerg Microbes Infect. 2018 Sep 19;7:157. doi: 10.1038/s41426-018-0156-4 (PMC6143531; doi:10.1038/s41426-018-0156-4)
Supplement: Supplementary file 3 — Supplementary Table S2 [file 41426_2018_156_MOESM3_ESM.docx]

**Table S2.** Molecular analyses (PCR and sequencing) performed on blood samples, skin tissue samples, and lesion swabs (RKI). P=positive PCR result or sequences generated but too short to be uploaded into EMBL; N=negative PCR result. * clinically affected individual. ^$^ samples from which genome sequencing was successful.

| **Species** | **Group** | **ID** | **Sample type** | ***TP polA*** |
| --- | --- | --- | --- | --- |
| *Cercocebus atys* | TCP | HATO^*,$^ | face lesion biopsy | P |
|  |  |  | normal skin biopsy | N |
|  |  |  | blood | N |
|  |  | IGU^*,$^ | face lesion biopsy | P |
|  |  |  | arm lesion biopsy | P |
|  |  |  | normal skin biopsy | N |
|  |  | KAH | normal skin biopsy | N |
|  |  |  | blood | N |
|  |  | RAN | normal skin biopsy | N |
|  |  |  | blood | N |
|  |  | PHA* | face lesion biopsy | N |
|  |  |  | blood | N |
| *Chlorocebus sabaeus* | BFP | M2*^,$^ | face lesion biopsy | P |
|  |  |  | blood | N |
|  |  | M3*^,$^ | face lesion biopsy | P |
|  |  |  | normal skin biopsy | N |
|  |  |  | blood | N |
|  |  | M7* | face lesion biopsy | P |
|  |  |  | genital lesion biopsy | N |
|  |  |  | blood | N |
|  |  | M6 | normal skin biopsy | N |
|  |  |  | blood | N |
|  |  | M4 | normal skin biopsy | N |
|  |  |  | blood | N |
