# Supplementary material for: Nonhuman primates across sub-Saharan Africa are infected with the yaws bacterium Treponema pallidum subsp. pertenue
Source: Emerg Microbes Infect. 2018 Sep 19;7:157. doi: 10.1038/s41426-018-0156-4 (PMC6143531; doi:10.1038/s41426-018-0156-4)
Supplement: Supplementary file 4 — Supplementary Table S3 [file 41426_2018_156_MOESM4_ESM.docx]

**Table S3.** Read mapping and genotyping results (EKU). Read mapping and genotyping results using EAGER. Here, details on raw reads, mapped reads, mean coverage of Fribourg-Blanc genome, percentage of genome that is covered by at least three reads and number of SNPs are shown. Note that EAGER outputs a more extensive result table, including percentage of endogenous DNA, duplication factors and many more. The full report table is available as supplementary material.

| Strain  (Host species, ID) | No. of raw reads | Mapped reads after duplicate removal | Mean coverage | Coverage ≥ 3X [%] | # of SNPs |
| --- | --- | --- | --- | --- | --- |
| TaïNP-1  (*Cercocebus atys*, IGU) | 3.56E+5 | **2.29E+4** | 6.12 | 82.39 | 207 |
| TaïNP-2  (*Cercocebus atys*, HATO) | 1.28E+6 | **1.08E+5** | 29.64 | 99.90 | 228 |
| Gambia-1  (*Chlorocebus sabaeus*, M2) | 6.62E+6 | **4.19E+5** | 113.12 | 99.99 | 101 |
| Gambia-2  (*Chlorocebus sabaeus*, M3) | 5.74E+6 | **4.47E+5** | 121.26 | 100.00 | 102 |
| NKNP-1  (*Chlorocebus sabaeus*, A10) | 7.49E+6 | **4.70E+5** | 114.71 | 99.96 | 115 |
| NKNP-2  (*Chlorocebus sabaeus*, A12) | 3.17E+6 | **2.07E+5** | 46.71 | 96.53 | 109 |
| LMNP-1  (*Papio anubis*, BS1-40M5160407) | 4.33E+7 | 5.07E+4 | 5.27 | 75.08 | 158 |
| LMNP-1  (*Papio anubis*, BS2-40M5160407) | 1.19E+8 | 1.42E+5 | 15.07 | 98.18 | 208 |
| LMNP-1  (*Papio anubis*, BS3-40M5160407) | 2.75E+7 | 3.65E+4 | 3.98 | 56.58 | 127 |
| LMNP-1  (*Papio anubis*, BS4-40M5160407) | 3.44E+7 | 8.30E+3 | 0.94 | 8.86 | 29 |
| LMNP-1  (*Papio anubis*, 40M5160407[-pooled]) | - | **2.40E+5** | 25.85 | 99.80 | 244 |
| LMNP-2  (*Papio anubis*, BS4-4F5230307) | 2.83E+7 | 2.14E+4 | 2.20 | 23.84 | 46 |
| LMNP-2  (*Papio anubis*, BS5-4F5230307) | 2.99E+7 | 2.62E+4 | 2.67 | 29.99 | 71 |
| LMNP-2  (*Papio anubis*, BS6-4F5230307) | 1.85E+7 | 1.73E+4 | 1.76 | 16.83 | 36 |
| LMNP-2  (*Papio anubis*, BS7-4F5230307) | 3.99E+7 | 3.75E+4 | 3.85 | 48.12 | 103 |
| LMNP-2  (*Papio anubis*, BS8-4F5230307) | 3.49E+7 | 1.31E+3 | 0.12 | 0.45 | 0 |
| LMNP-2  (*Papio anubis*, 4F5230307[-pooled]) | - | **7.78E+4** | **7.97** | 88.70 | 174 |
