# Supplementary material for: Nonhuman primates across sub-Saharan Africa are infected with the yaws bacterium Treponema pallidum subsp. pertenue
Source: Emerg Microbes Infect. 2018 Sep 19;7:157. doi: 10.1038/s41426-018-0156-4 (PMC6143531; doi:10.1038/s41426-018-0156-4)
Supplement: Supplementary file 5 — Supplementary Table S4 [file 41426_2018_156_MOESM5_ESM.docx]

**Table S4.** Published genomes used for phylogenetic analyses.

| **Sample** | **RefSeq ID** |
| --- | --- |
| Nichols | NC_021490.2 |
| SS14 | NC_021508.1 |
| Chicago | NC_017268.1 |
| Mexico A | NC_018722.1 |
| Dallas | NC_016844.1 |
| Seattle 81-4 | CP003679.1 |
| Fribourg-Blanc | NC_021179.1 |
| Samoa D | NC_016842.1 |
| CDC-2 | NC_016848.1 |
| Gauthier | NC_016843.1 |
| Bosnia A | CP007548.1 |
| CDC-2575 | SRR5320460 |
| GHANA-051 | SRR5319489 |
| Solomon Islands 03 | ERR1470342 |
| Solomon Islands 17 | ERR1470334 |
| Solomon Islands 20 | ERR1470338 |
| Solomon Islands 28 | ERR1470344 |
| Solomon Islands 30 | ERR1470343 |
| Solomon Islands 32 | ERR1470335 |
| Solomon Islands 37 liq | ERR1470330 |
| Solomon Islands 37 sca | ERR1470331 |
