# Supplementary material for: Nonhuman primates across sub-Saharan Africa are infected with the yaws bacterium Treponema pallidum subsp. pertenue
Source: Emerg Microbes Infect. 2018 Sep 19;7:157. doi: 10.1038/s41426-018-0156-4 (PMC6143531; doi:10.1038/s41426-018-0156-4)
Supplement: Supplementary file 6 — Supplementary Table S5 [file 41426_2018_156_MOESM6_ESM.docx]

**Table S5.** List of primers used for long-range PCR amplification of *TP* intervals of the East African baboon genome (strain LMNP-1).

| **POOL^a^** | **TP-interval** | **Primer pair** | **Primer orientation** | **Primer sequence (5'->3')^b^** | **Primer coordinates^b^** | | **Primer length (nt)^b^** | **PCR product length (nt)^b^** |
| --- | --- | --- | --- | --- | --- | --- | --- | --- |
| POOL-1 | TPI-1A | ES-154R | **F**orward | CGGCTGTATTTCGTTACTGTCTTGA | 1135699 | 1135723 | 25 | 5931 |
|  |  | 1A_newR | **R**everse | CTGCGACATCCTCAGTACCA | 3599 | 3618 | 20 |  |
| POOL-1 | TPI-1B | 1B_newF | F | TTTGATCTCGTGCAGTGGAG | 3313 | 3332 | 20 | 6074 |
|  |  | ES-72R_new | R | GAACTCGCCGGTAATGCTAC | 9367 | 9386 | 20 |  |
| POOL-1 | TPI-2A-A | ES-71F | F | TCTCCCGAACGAGTTTCTAGTCTG | 8920 | 8943 | 24 | 6599 |
|  |  | 2A-A_newR | R | AGTGATTGTTGCGTGCATGT | 15499 | 15518 | 20 |  |
| POOL-1 | TPI-2A-B1 | 2A-B-1-F | F | TCTCCATGCGGTAGATCTGATT | 15108 | 15129 | 22 | 2501 |
|  |  | 2A-B-1-R | R | CAGCGCGTTAATTCTCCCTATT | 17587 | 17608 | 22 |  |
| POOL-1 | TPI-2A-B2 | 2A-B-2-F | F | AGAACTGGCAGAGAGTCATACA | 17356 | 17377 | 22 | 2501 |
|  |  | 2A-B-2-R | R | TCCCTTCCTGCTCGTATTTATC | 19835 | 19856 | 22 |  |
| POOL-1 | TPI-2A-B3 | 2A-B-3-F | F | CGTGAAAGTGAGGGTGAAGTA | 19347 | 19367 | 21 | 2482 |
|  |  | 2A-B-3-R | R | CAAACGAAACTCTGCCAGCTAC | 21807 | 21828 | 22 |  |
| POOL-1 | TPI-2B | TPI-2BF_new | F | AACAAGCAGGCTCTTTCGGTA | 21358 | 21377 | 20 | 6962 |
|  |  | TPI-2B_newR2 | R | GCGGTATGTCCACCGTATCT | 28300 | 28319 | 20 |  |
| POOL-1 | TPI-3A-A | TPI-3-AF-Bosnia | F | TCGTAGGAAACAGGGACGAG | 27562 | 27581 | 20 | 4731 |
|  |  | 3A-A_newR | R | TATCACCCGTCAAACTGCAA | 32273 | 32292 | 20 |  |
| POOL-1 | TPI-3A-B | 3A-A_newF | F | CGGGGATATAACGCAAAGAA | 31653 | 31672 | 20 | 4534 |
|  |  | TPI-3-AR-Bosnia | R | AGTGTACGAAACCCCAGACG | 36167 | 36186 | 20 |  |
| POOL-1 | TPI-3B | TPI-3-BF-Bosnia | F | GTGCTCTCTAGCGTGGTGAC | 35661 | 35680 | 20 | 6892 |
|  |  | TPI-3-BR-Bosnia | R | ATGGGGTTGACTCTCACGAC | 42533 | 42552 | 20 |  |
| POOL-1 | TPI-4A-1 | 4A_newF | F | GCCAGAGCAGCATAAGGAAC | 41511 | 41530 | 20 | 4838 |
|  |  | 4A_newR1 | R | TCATACTCGTCCGTGCTCTG | 46329 | 46348 | 20 |  |
| POOL-1 | TPI-4B-A | TPI_4A-4B-F | F | CGAGAAAGACATGGACAGCA | 46143 | 46162 | 20 | 5015 |
|  |  | TPI-4B-AR-Bosnia | R | ACCGAATTTGTAGCGTACCG | 51138 | 51157 | 20 |  |
| POOL-1 | TPI-4B-B | TPI-4B-BF-Bosnia | F | GAGTCCACTACAGCGTCTCG | 50789 | 50808 | 20 | 3471 |
|  |  | TPI-4B-BR-Bosnia | R | TAGCGTATCTCCACCCCAAC | 54240 | 54259 | 20 |  |
| POOL-1 | TPI-5A-A | TPI-5A-AF-Bosnia | F | TGTATGCCCAGAAGGTGAAG | 52900 | 52919 | 20 | 7247 |
|  |  | TPI-5A-AR-Bosnia | R | TCAGTGCTAGGGACACGTTG | 60127 | 60146 | 20 |  |
| POOL-1 | TPI-5A-B | TPI-5A-BF-Bosnia | F | ATCCAAGATGAGTCGGATGC | 59770 | 59789 | 20 | 5920 |
|  |  | TPI-5A-BR-Bosnia | R | AGAATGTGGGAGCGATCTTG | 65670 | 65689 | 20 |  |
| POOL-1 | TPI-5B-A | TPI-5B-F | F | CGCTACCACACCCTATCATACCG | 64800 | 64822 | 23 | 6541 |
|  |  | 5B-A_newR | R | GAAGCCCCTCATACAGGACA | 71321 | 71340 | 20 |  |
| POOL-1 | TPI-5B-B | 5B-B_newF | F | GCACAAACAAGTGCGTGAGT | 70718 | 70737 | 20 | 6611 |
|  |  | ES-119R | R | TGAAAGTCAGCCTGTTGAGCTAGAC | 77304 | 77328 | 25 |  |
| POOL-1 | TPI-6A-A | ES-118F | F | TCATCAAAAAGAAGTACGCTGT | 76498 | 76519 | 22 | 5050 |
|  |  | 6A-A_newR | R | TTGATGCGGTACTTGTGGAA | 81528 | 81547 | 20 |  |
| POOL-1 | TPI-6A-B | 6A-B_newF2 | F | GCCGCATTACGATTTTGAAG | 81374 | 81393 | 20 | 4295 |
|  |  | 6A-B_newR1 | R | GTCGGCTCTATACGCTCGTC | 85649 | 85668 | 20 |  |
| POOL-1 | TPI-6B-A | TPI-6BF | F | CCGTTACCGTGACAGATCCT | 85395 | 85414 | 20 | 3488 |
|  |  | 6B-A_newR | R | CACGCACAACGCATCTAATTC | 88862 | 88882 | 21 |  |
| POOL-1 | TPI-6B-B | 6B-B_newF (BIS) | F | GTTTGCGTGTCTGGAGGATGC | 88474 | 88494 | 21 | 2661 |
|  |  | 6B-B_newR (BIS) | R | CAGAAGACGTTGCGCTTCTAGC | 91113 | 91134 | 22 |  |
| POOL-1 | TPI-7A-A | TPI-7AF | F | GCGCCTGATGAAAGTTGACC | 90757 | 90776 | 20 | 5972 |
|  |  | 7A-A_newR | R | TTACTGGGGTTGCTTTCCTG | 96709 | 96728 | 20 |  |
| POOL-1 | TPI-7A-B | 7A-B_newF | F | AGCCGAGTACGAGCATGAGT | 95987 | 96006 | 20 | 5562 |
|  |  | TPI-7AR | R | CACGCGCTTCCAAATAGTCC | 101529 | 101548 | 20 |  |
| POOL-1 | TPI-7B-A1 | 7B-A1_F (BIS) | F | TTACCGAGTGAGGAGTGGATTGG | 101393 | 101415 | 23 | 3239 |
|  |  | 7B-A1_R (BIS) | R | AATGCGCTTGCTGCACGATAC | 104611 | 104631 | 21 |  |
| POOL-1 | TPI-7B-A2 | 7B-A2_F | F | TTGCGCAGTATCCAGACAATAAG | 104400 | 104422 | 23 | 3494 |
|  |  | TPI-7B-AR-Bosnia | R | ATCAGGAAGACAGGGAGCTG | 107874 | 107893 | 20 |  |
| POOL-1 | TPI-7B-B | TPI-7B-BF-Bosnia | F | CACGAACTCTTGCCTGTTCA | 107391 | 107410 | 20 | 6205 |
|  |  | TPI-7B-BR-Bosnia | R | AGGACATCACCCCACAAGAG | 113576 | 113595 | 20 |  |
| POOL-1 | TPI-8 | ES-122F | F | GATTCTGCCGTTTTGGTAACTACTTG | 113063 | 113088 | 26 | 5053 |
|  |  | ES-162F | R | TAACTCATGCCCAACAGTAGCTTG | 118092 | 118115 | 24 |  |
| POOL-1 | TPI-9 | ES-163R | F | GGATTGCTTTCTGTGTTTGAGACC | 117579 | 117602 | 24 | 5351 |
|  |  | ES-151F | R | TTCACAGATTAACGGCATTGGAC | 122907 | 122929 | 23 |  |
| POOL-1 | TPI-10A | ES-152R | F | ACCGCGGAACGAATCAAGTAG | 122504 | 122524 | 21 | 5722 |
|  |  | 10A_newR | R | GCGGAGAACTCGGGTAATTC | 128206 | 128225 | 20 |  |
| POOL-1 | TPI-10B | 10A_newF | F | AGTGTGTGTCGAACGCTCAG | 127725 | 127744 | 20 | 6040 |
|  |  | ES-43R | R | CAATACCCCATCTCTCCGAGC | 133744 | 133764 | 21 |  |
| POOL-4 | TPI-11A | ES-42F | F | TTATCAGCCTGAATCGTATGTCCC | 133144 | 133167 | 24 | 4835 |
|  |  | TPI-11A-R | R | AGAGCCATTGTTGCAGTCTC | 137959 | 137978 | 20 |  |
| POOL-1 | TPI-11B | TPI-11B-F | F | TCAGTGTCCTGTACGCCAAG | 137894 | 137913 | 20 | 4509 |
|  |  | TPI-11B-R | R | GGGTACTGATGGTGAGTTCG | 142383 | 142402 | 20 |  |
| POOL-1 | TPI-11C | TPI-11C-F | F | TGGCGTTTTCCTTGATAGAG | 141882 | 141901 | 20 | 3995 |
|  |  | ES-35F | R | ATGTGCAAGTACTCCAACCTCTCG | 145853 | 145876 | 24 |  |
| POOL-1 | TPI-12A-1 | ES-36R | F | GGTTGGAGTACTTGCACATGTGG | 145858 | 145880 | 23 | 3127 |
|  |  | 12R-3127 | R | CGTTGCACTACACTCGATTC | 148965 | 148984 | 20 |  |
| POOL-1 | TPI-12A-3 | 12F-2761 | F | GTCCAAAAGTGTGATGCTGT | 148618 | 148637 | 20 | 3892 |
|  |  | TPI-12AF | R | CCAGTCCTTCCTGTGTGGTT | 152490 | 152509 | 20 |  |
| POOL-1 | TPI-12B | TP0131-XL-F | F | AAGCTAAACGAGCCTCCACA | 150275 | 150294 | 20 | 5421 |
|  |  | ES-50F | R | TCATACGTTTTTTCGCTCCACAC | 155673 | 155695 | 23 |  |
| POOL-1 | TPI-13 | ES-51R | F | TCTGCCTTACAGGTGACGTCCT | 154900 | 154921 | 22 | 4761 |
|  |  | ES-25F | R | CTCCAATACACGAAACTATCTTGCG | 159636 | 159660 | 25 |  |
| POOL-1 | TPI-14A-A | TPI-14A-AF-Bosnia | F | GTTAATTCCCGCAAATCACG | 159288 | 159307 | 20 | 6495 |
|  |  | TPI-14A-AR-Bosnia | R | ATGCGTGTTGTCTTCTGCTG | 165763 | 165782 | 20 |  |
| POOL-1 | TPI-14A-B | TPI-14A-BF-Bosnia | F | ACGTTAGTATGCGGGTCTGC | 165277 | 165296 | 20 | 6847 |
|  |  | TPI-14A-BR-Bosnia | R | GCTCCTTTGTTCGAGGTCTG | 172104 | 172123 | 20 |  |
| POOL-1 | TPI-14B-A1 | TPI-14B-AF1-Bosnia | F | ACCGTCAGTGGGAGTTGTTC | 170987 | 171006 | 20 | 4102 |
|  |  | TPI-14B-AR1-Bosnia | R | GGTAAAGGTTGGGGATACGG | 175069 | 175088 | 20 |  |
| POOL-1 | TPI-14B-A2 | TPI-14B-AF2-Bosnia | F | GAACTCAAAGGGCAGGTAG | 174579 | 174597 | 19 | 7028 |
|  |  | TPI-14B-AR2-Bosnia | R | CCGGAGTAAAGTTCGCAGAC | 181587 | 181606 | 20 |  |
| POOL-1 | TPI-14B-B1 | TPI-14B-BF-Bosnia | F | TTCTCGTTCATTGTGCTCGT | 178698 | 178717 | 20 | 2917 |
|  |  | SDTP0159-0160R | R | AGAGCGACCCGGAGTAAAGT | 181595 | 181614 | 20 |  |
| POOL-1 | TPI-14B-B2 | SDTP0159-0160L | F | GGACAAAAGAAGCGAACTGC | 180859 | 180878 | 20 | 4582 |
|  |  | TPI-14B-BR-Bosnia | R | AACAACTCGCCTCCAATCTG | 185421 | 185440 | 20 |  |
| POOL-1 | TPI-15A-A | ES-113R | F | TGTATGTCGTGCAGTTCTAGCCC | 183634 | 183656 | 23 | 4842 |
|  |  | TPI-15A-1R | R | CGACATCGACTCAGCCTACA | 188456 | 188475 | 20 |  |
| POOL-1 | TPI-15A-B | TPI-15A-2F | F | GTTCGCGTTGGTGTACCTTT | 188148 | 188167 | 20 | 4596 |
|  |  | TP-15AR | R | ATGGTCTTAACGGTGGCTTG | 192724 | 192743 | 20 |  |
| POOL-1 | TPI-15B-A | TP-15BF | F | GAACTCCACGAGCTTTTTCG | 191747 | 191766 | 20 | 4312 |
|  |  | TPI-15B-1R | R | GGATGGCAAACCTGTTCACT | 196039 | 196058 | 20 |  |
| POOL-1 | TPI-15B-B | TPI-15B-2F | F | CCACTGGATATCGGATGAAG | 195509 | 195528 | 20 | 4988 |
|  |  | TPI-15B-2R | R | CTCGGCTGCTTATCCTGTTC | 200477 | 200496 | 20 |  |
| POOL-1 | TPI15B-C | TPI-15B-3F | F | CACAGGACATCGACCACATC | 199465 | 199484 | 20 | 3572 |
|  |  | TPI-15B-3R | R | ACCCATTCTCACGACCACTC | 203017 | 203036 | 20 |  |
| POOL-1 | TPI15B-D | 15B-D_newF | F | GAACAGGGACTGGATGTGGT | 202821 | 202840 | 20 | 2914 |
|  |  | 15B-D_newR | R | ATAAATCACACCCCGGTTCA | 205715 | 205734 | 20 |  |
| POOL-1 | TPI-16A-A | TPI-16A-AF-Bosnia | F | TCTGCGTATTGAACGGATTG | 205508 | 205527 | 20 | 6649 |
|  |  | TPI-16A-AR-Bosnia | R | CGCACAATGGTCTCGTAATC | 212137 | 212156 | 20 |  |
| POOL-1 | TPI-16A-B | TPI-16A-BF-Bosnia | F | TTACGGTTCGTGTTGAGCAG | 211766 | 211785 | 20 | 6403 |
|  |  | TPI-16A-BR-Bosnia | R | TGCTCCTGCAAAACATTCAC | 218149 | 218168 | 20 |  |
| POOL-1 | TPI-16B-A | 16B-A_newF | F | AAAGGAGCTGGGAAGAAGGA | 217456 | 217475 | 20 | 6006 |
|  |  | 16B-A_newR | R | CCATTACATCCCCAGGACAC | 223442 | 223461 | 20 |  |
| POOL-1 | TPI-16B-B | 16B-B_newF | F | AATTGACACAGCGGGTATGA | 223277 | 223296 | 20 | 6199 |
|  |  | 16B-B_newR | R | ACCGAACTAGGCAACGACAC | 229456 | 229475 | 20 |  |
| POOL-1 | TPI-17A-A2 | ES-45R | F | GAAGCAGAATGCTGTCTCTCGTG | 229251 | 229273 | 23 | 7888 |
|  |  | TPI-17A-AR-Bosnia | R | AGCCTGTTCCGCTTGTACTG | 237119 | 237138 | 20 |  |
| POOL-1 | TPI-17A-B | TPI-17A-BF-Bosnia | F | CTGTAGGGGCAAGATGATGG | 236671 | 236690 | 20 | 6688 |
|  |  | TPI-17A-BR-Bosnia | R | CAGAATAGACGGTGACAACCA | 243338 | 243358 | 21 |  |
| POOL-1 | TPI-17B1-A | 17B1-A_newF | F | ACAGGTGAGGATCGTTGAGG | 242906 | 242925 | 20 | 3475 |
|  |  | 17B1-A_newR | R | TTTCACCCTGCTCATTTTCC | 246361 | 246380 | 20 |  |
| POOL-1 | TPI-17B1-B | 17B1-B_newF | F | GGCGCTTGTTGATTTACGTT | 245917 | 245936 | 20 | 3942 |
|  |  | 17B1-B_newR | R | ACCATAGGACGCAAATCAGG | 249839 | 249858 | 20 |  |
| POOL-1 | TPI-17B2 | TPI-17B2R | F | ACTGAGCGCGATGAAGAAAT | 249074 | 249093 | 20 | 7411 |
|  |  | ES-77F | R | ATGGCGTACGTAGGTCCGTGT | 256464 | 256484 | 21 |  |
|  |  |  |  |  |  |  |  |  |
| POOL-2 | TPI-19 | ES-78R | F | GCGCAGTAAAAGAGGGACGAC | 255635 | 255655 | 21 | 6413 |
|  |  | ES-56F | R | CATCATCACGTACAAACCATGAGG | 262024 | 262047 | 24 |  |
| POOL-2 | TPI-20A | ES-57R | F | CCTCTCGCGTTATTGAGGCTC | 261725 | 261745 | 21 | 4351 |
|  |  | 20A_newR | R | GGCGAATCTGACTTGGAGAG | 266056 | 266075 | 20 |  |
| POOL-2 | TP-20B-A | 20B_newF | F | GGTGTCGGTAATCTCGGTGT | 265490 | 265509 | 20 | 3029 |
|  |  | N+D_TP0256R | R | CGGAGGAGGGAAAGTTCTTC | 268499 | 268518 | 20 |  |
| POOL-2 | TP-20B-B | N+D_TP0256F | F | TTGAAAGCATTGTACGCCTTT | 267890 | 267910 | 21 | 2328 |
|  |  | ES-75F | R | TACTAAGCTTCCTCATCATCGTCTCC | 270192 | 270217 | 26 |  |
| POOL-2 | TPI-21A-A | 21A-F | F | GCTTACCCTCGCTCTGTTGA | 268732 | 268751 | 20 | 3948 |
|  |  | 21A-A-R | R | CGAGAGAACACCTTGAGCATCTTC | 272656 | 272679 | 24 |  |
| POOL-2 | TPI-21A-B | 21A-A-F | F | AGCCATTCAGTATAAGGCCAATTC | 272374 | 272397 | 24 | 3899 |
|  |  | 21A-R | R | GATAAAATCCGCGTGTCCTG | 276253 | 276272 | 20 |  |
| POOL-2 | TPI-21C-A | 21C-F | F | CGCGTCGGTATGTGTAAATC | 275915 | 275934 | 20 | 2302 |
|  |  | 21C-A_newR | R | TGCAGGAAACCTCATCCTTC | 278197 | 278216 | 20 |  |
| POOL-2 | TPI-21C-B | 21C-B_newF | F | TGCGATTGGTATGAGGACTG | 278111 | 278130 | 20 | 6187 |
|  |  | 21C-B_newR | R | GGGCACACGCAATGTAGTCT | 284278 | 284297 | 20 |  |
| POOL-2 | TPI-21C-C | 21C-C_newF | F | AAGCTATCGCACAGGGGTCT | 284076 | 284095 | 20 | 4067 |
|  |  | 21C-R | R | CAGCGAGAAAACCTGAATCC | 288123 | 288142 | 20 |  |
| POOL-2 | TPI-21D | 21D_newF | F | ACGAAAGCGACTGCTGAACT | 287971 | 287990 | 20 | 2573 |
|  |  | 21D_newR | R | GTGACGAACATGGACACCAG | 290524 | 290543 | 20 |  |
| POOL-2 | TPI-23A-A | ES-109R | F | GTTTCGAACTGGAAAATACGTGTGTAC | 289715 | 289741 | 27 | 5427 |
|  |  | 23A-A_newR | R | CCCTCCTGATTTTCCACTGA | 295122 | 295141 | 20 |  |
| POOL-2 | TPI-23A-B | 23A-B_newF2 | F | GACGGTTGTGTTTGTGGATG | 294842 | 294861 | 20 | 4856 |
|  |  | 23A-B_newR2 | R | TCGCACAAATCACGCACTAC | 299678 | 299697 | 20 |  |
| POOL-2 | TPI-23B-A | TPI-23BF | F | TGCTTCACTGATGCCAAAAG | 299479 | 299498 | 20 | 4575 |
|  |  | 23B-A_newR | R | TTGCCCGTCTTAATTTCACG | 304034 | 304053 | 20 |  |
| POOL-2 | TPI-23B-B | 23B-B_newF | F | GCACAGGTTAGGTTGGTGGT | 303821 | 303840 | 20 | 4393 |
|  |  | ES-147F | R | GACAAGCGAGCGTGTAGGAGTC | 308192 | 308213 | 22 |  |
| POOL-2 | TPI-24A-A | TPI_23-24A-F | F | GCGGAGGTACGATGCTAAAG | 307895 | 307914 | 20 | 4899 |
|  |  | TPI-24A-AR-Bosnia | R | CGGATTGCGAAACAATACAC | 312774 | 312793 | 20 |  |
| POOL-2 | TPI-24A-B1 | TPI-24A-BF-Bosnia-new | F | AGGAGCGTCTTGCGTATGAG | 311951 | 311970 | 20 | 2902 |
|  |  | TPI-24A-innerB-R-Bosnia | R | AGCACCACATTTACCGTCAG | 314833 | 314852 | 20 |  |
| POOL-2 | TPI-24A-B2 | TPI-24A-innerB-F-Bosnia | F | TCTGTAGTTGGGCTGTTTGG | 314662 | 314681 | 20 | 3346 |
|  |  | TPI-24A-BR-Bosnia-new | R | GGTTCAGTACGGCTTCCAAC | 317988 | 318007 | 20 |  |
| POOL-2 | TPI-24B-A | TPI-24(B)_newF | F | CCAGTGGAGCGGCACCGCAGTTAC | 316219 | 316242 | 24 | 4947 |
|  |  | 24B-A_newR | R | TCTTGCGTGACAAACACCTC | 321146 | 321165 | 20 |  |
| POOL-2 | TPI-24B-B | 24B-B_newF | F | GCAACCCCTTCGGTAAAGAG | 320420 | 320439 | 20 | 4819 |
|  |  | ES-49R | R | CCGTGTGGTTCAAATCCAAACT | 325217 | 325238 | 22 |  |
| POOL-4 | TPI-25A | ES-26F | F | TTCCGAGCCATATCTGCGTACT | 325002 | 325023 | 22 | 5764 |
|  |  | TPI-25AR | R | CGTTTAATGTTCTGCGGCCGGTGTTTG | 330739 | 330765 | 27 |  |
| POOL-2 | TPI-25B-A | TPI-25(B)newF | F | GGGCGCCTTCCGGCAGGACTCT | 330289 | 330310 | 22 | 2001 |
|  |  | Nich323-R3 | R | TGTGCTGCTTCTGGTTATGC | 332270 | 332289 | 20 |  |
| POOL-2 | TPI-25B-B | Nich323-F2 | F | GAAGACGCAAGCTCTACTGC | 332036 | 332055 | 20 | 3821 |
|  |  | ES-47R | R | GGAACCACTATCTCCTTCGAGACAA | 335832 | 335856 | 25 |  |
| POOL-2 | TPI-25B | TPI-25(B)newF | F | GGGCGCCTTCCGGCAGGACTCT | 330289 | 330310 | 22 | 5568 |
|  |  | ES-47R | R | GGAACCACTATCTCCTTCGAGACAA | 335832 | 335856 | 25 |  |
| POOL-2 | TPI-26A-1 | TPI-26-AF-Bosnia | F | GTCGATCCCGACATACAGGT | 335396 | 335415 | 20 | 2554 |
|  |  | SDTP0321-0322R | R | CCTTCGCCCATATACCTGAA | 337930 | 337949 | 20 |  |
| POOL-2 | TPI-26A-2 | SDTP0321-0322L | F | GTGTTCTGCGTGGTGTCAGT | 337161 | 337180 | 20 | 4464 |
|  |  | SDTP0324-0325R | R | CATCACGTTCCCCTGTACCT | 341605 | 341624 | 20 |  |
| POOL-2 | TPI-26A-3 | SDTP0324-0325L | F | ACCCCGTATGTAGTGCTTGC | 340891 | 340910 | 20 | 2392 |
|  |  | TPI-26-AR-Bosnia | R | GGCGTTATACGAAGCTCCAG | 343263 | 343282 | 20 |  |
| POOL-2 | TPI-26B | TPI-26-BF-Bosnia | F | ATGGTTCAGTGTCGTTGCAG | 342763 | 342782 | 20 | 7804 |
|  |  | TPI-26-BR-Bosnia | R | CTGACACCCGCCATAGTAGC | 350547 | 350566 | 20 |  |
| POOL-2 | TPI-27A-A | ES-101F | F | CCTTGTTATCATGGACGAAGTAGGAC | 349567 | 349592 | 26 | 4621 |
|  |  | 27A-A_newR | R | TAGCCTGGTTTGATGGGTTC | 354168 | 354187 | 20 |  |
| POOL-2 | TPI-27A-B | 27A-A_newF | F | GTGCCTGTCCAGTTTGTGTG | 354049 | 354068 | 20 | 4388 |
|  |  | TPI-27AR | R | AGTTTTTGGGGTTGTGCAAG | 358417 | 358436 | 20 |  |
| POOL-2 | TPI-27B-A | TPI-27B-AF-Bosnia | F | CCATGACAGCAAGTCCAATG | 355439 | 355458 | 20 | 5028 |
|  |  | TPI-27B-AR-Bosnia | R | GATGCTCAGTTGCTCTGCAC | 360447 | 360466 | 20 |  |
| POOL-2 | TPI-27B-B | TPI-27B-BF-Bosnia | F | ATCGGGTTGAGAATGACTGC | 360107 | 360126 | 20 | 5867 |
|  |  | TPI-27B-BR-Bosnia | R | AGTTCACTCGGGTCATCAGG | 365954 | 365973 | 20 |  |
| POOL-2 | TPI-28A-A | ES-103F | F | TGAAATGTTACGCGCTAGAGGG | 365594 | 365615 | 22 | 4284 |
|  |  | TPI-28A-1R | R | GTGGCATACTCCATTCTTGG | 369858 | 369877 | 20 |  |
| POOL-2 | TPI-28A-B | TPI-28A-2F | F | CGCTATACGCTCCTCACTCC | 369165 | 369184 | 20 | 4531 |
|  |  | TPI-28A-2R | R | TGGGACGCTCTTTTCTTATG | 373676 | 373695 | 20 |  |
| POOL-2 | TPI-28A-C | TPI-28A-3F | F | ACAAGGTGTGAGGGAGTTGG | 371973 | 371992 | 20 | 3697 |
|  |  | TPI-28A-3R | R | ATGTGCCTGAGTGCTTCTTC | 375650 | 375669 | 20 |  |
| POOL-2 | TPI-28A-D | 28A-D_newF | F | CATGCAATTCAGTCGAAACG | 375396 | 375415 | 20 | 3498 |
|  |  | 28A-D_newR | R | AGAGAAACACCCGTCACACC | 378874 | 378893 | 20 |  |
| POOL-2 | TPI-28B-A | TPI-28B-AF-Bosnia | F | ACGTATTGGGAGTCGGTGAC | 377436 | 377455 | 20 | 6234 |
|  |  | TPI-28B-AR-Bosnia | R | AACACCCTCTGCTACGCACT | 383650 | 383669 | 20 |  |
| POOL-2 | TPI-28B-B | TPI-28B-BF-Bosnia | F | TCCAACGCGCATACAACTAC | 383152 | 383171 | 20 | 6100 |
|  |  | TPI-28B-BR-Bosnia | R | TATAGTTCTTCGCGCCCAGT | 389232 | 389251 | 20 |  |
| POOL-2 | TPI-29A-A | ES-33F | F | CAGATTGGTCAAATCTTGACAAGTGA | 388990 | 389015 | 26 | 5919 |
|  |  | 29A-A_newR | R | GAGACAAAGAATCCGCAAGC | 394889 | 394908 | 20 |  |
| POOL-2 | TPI-29A-B | 29A-B_newF | F | GAAAAACGCGATCACCTAGC | 394031 | 394050 | 20 | 5951 |
|  |  | TP-29AR | R | GTTACGCGGGTCTTTGGTAA | 399962 | 399981 | 20 |  |
| POOL-2 | TPI-29B-A | 29B-A_newF | F | GAGTATGGAGCGAAGGGACA | 399772 | 399791 | 20 | 3174 |
|  |  | 29B-A_newR2 | R | ATTACGCACTGGGAGGTGAG | 402926 | 402945 | 20 |  |
| POOL-2 | TPI-29B-B | 29B-B_newF2 | F | TGTATTCGGTAAGCGGGTTC | 402682 | 402701 | 20 | 3176 |
|  |  | 29B-B_newR | R | GTGCCGTGGAAGGATTTAGA | 405838 | 405857 | 20 |  |
| POOL-2 | TPI-29C | 29C-F | F | CCGCTACTTTTGCTTCGTTC | 405701 | 405720 | 20 | 4899 |
|  |  | 29C-R | R | GCGACTGCGGTAAACCTATC | 410580 | 410599 | 20 |  |
| POOL-2 | TPI-30A-A | ES-69R | F | GTACAATCTCGATGAAAAGGGGC | 408201 | 408223 | 23 | 4983 |
|  |  | TPI-30A-1R | R | AGAGGAACAAGCTGATCGTG | 413164 | 413183 | 20 |  |
| POOL-2 | TPI-30A-B | TPI-30A-2F | F | GAGGGAGCGTGAAGAAACAG | 412625 | 412644 | 20 | 5081 |
|  |  | TPI-30AF | R | GAGCAATCTTGTGCGTTTCA | 417686 | 417705 | 20 |  |
| POOL-2 | TPI-30B-A | TPI-30BR | F | CACACGTGGAAAATGGACAG | 416848 | 416867 | 20 | 4780 |
|  |  | TPI-30B-1R | R | ACATATCGCTGGGTGGTAGA | 421608 | 421627 | 20 |  |
| POOL-2 | TPI-30B-B | TPI-30B-2F | F | CACAGTTTTGCTTCGACGTT | 421530 | 421549 | 20 | 3663 |
|  |  | TPI-30B-2R | R | ACTGCATCCTTGAGTTCTGC | 425173 | 425192 | 20 |  |
| POOL-2 | TPI-30B-C | TPI-30B-3F | F | ATACTCCCTTGCCAGTGTGG | 424682 | 424701 | 20 | 4229 |
|  |  | ES-80R | R | ACATGGACCGCCTTCCTCATA | 428890 | 428910 | 21 |  |
| POOL-2 | TPI-31A-A | TPI-31A-AF-Bosnia | F | CAGCGTGAGATTGGGTTAGC | 427830 | 427849 | 20 | 5469 |
|  |  | TPI-31A-AR-Bosnia | R | TGCCCCTTGTATTCTTCCAC | 433279 | 433298 | 20 |  |
| POOL-2 | TPI-31A-B | TPI-31A-BF-Bosnia | F | TTGAGCACCGTATGAAGCAG | 433013 | 433032 | 20 | 5395 |
|  |  | TPI-31A-BR-Bosnia | R | GGCGATGTAAATGGTCGAGT | 438388 | 438407 | 20 |  |
| POOL-2 | TPI-31B-A1 | TPI-31(B)newF | F | GTGCGTCAGGCGTTGTTGTTGGTTTTG | 437958 | 437984 | 27 | 3110 |
|  |  | MexATP0414-0415R | R | AACGCACTCACCTCACCACT | 441048 | 441067 | 20 |  |
| POOL-2 | TPI-31B-A2 | MexATP0414-0415F | F | GTCGTAGTTCCTCGCGTAGC | 440397 | 440416 | 20 | 2747 |
|  |  | SDTP0415-0416R | R | TATGGGGGACATCGTTTCTC | 443124 | 443143 | 20 |  |
| POOL-2 | TPI-31B-B1 | SDTP0415-0416L | F | TCTGTTGGTCGGTGATCGTA | 442346 | 442365 | 20 | 3005 |
|  |  | SDTP0417R | R | CCCTCTGGGAATGGGTAAAG | 445331 | 445350 | 20 |  |
| POOL-2 | TPI-31B-B2 | SDTP0417L | F | CATCTGCGCCAGTCTTTGTA | 444569 | 444588 | 20 | 3291 |
|  |  | ES-92R | R | ACTCAAACACCAACCCCCTCTC | 447838 | 447859 | 22 |  |
| POOL-2 | TPI-32A-A | ES-91F | F | CACTTTCCTCCGAGGACGTGT | 447397 | 447417 | 21 | 5274 |
|  |  | 32A-A_newR | R | TCAAACTCGCCCCATCTATC | 452651 | 452670 | 20 |  |
| POOL-2 | TPI-32A-B | 32A-B_newF | F | GGATTGTGCGCTACGTTTCT | 452548 | 452567 | 20 | 5504 |
|  |  | TPI-32(A)newR | R | GCGCCGCACGACGATCAAATGA | 458030 | 458051 | 22 |  |
| POOL-2 | TPI-32B-A1 (AF1AR4) | TPI-32B-AF1-Bosnia | F | TGAGTTGCAGACGCTTTGTC | 457148 | 457167 | 20 | 3806 |
|  |  | TPI-32B-AR4-Bosnia | R | GCAAAGATGCAAGGGACAGT | 460934 | 460953 | 20 |  |
| POOL-2 | TPI-32B-F7R10 | TPI-32B-F7 | F | ACACTGCTGTTGAAATTAGC | 460732 | 460751 | 20 | 1039 |
|  |  | TPI-32B-R10 | R | CGTAACGGTGGACAATGCTC | 461751 | 461770 | 20 |  |
| POOL-2 | TPI-32Brep (F1R1) | 32Brep-F1 | F | CGTTTGGTTTCCCCTTTGTC | 461058 | 461077 | 20 | 481 |
|  |  | 32Brep-R1 | R | GTGGGATGGCTGCTTCGTATG | 461518 | 461538 | 21 |  |
| POOL-2 | TPI-32B-B1 (BF1BR1) | TPI-32B-BF1-Bosnia | F | CTCGGTTTCCAACTCAGCTC | 461538 | 461557 | 20 | 3459 |
|  |  | TPI-32B-BR1-Bosnia | R | ATTTGTGTCTTCGGGCAGTC | 464977 | 464996 | 20 |  |
| POOL-2 | TPI-32B-C | TPI-32B-CF-Bosnia | F | TCAGGTCTCTGTGTGGATGC | 464480 | 464499 | 20 | 4740 |
|  |  | TPI-32B-CR-Bosnia | R | TCACCTCCAATTCCCACATC | 469200 | 469219 | 20 |  |
| POOL-2 | TPI-33A-A | TPI-33A-AF-Bosnia | F | GACAGCATGAACACCAATCG | 468181 | 468200 | 20 | 6893 |
|  |  | TPI-33A-AR-Bosnia | R | GCTCCTCGTAACATGACACA | 475054 | 475073 | 20 |  |
| POOL-2 | TPI-33A-B | TPI-33A-BF-Bosnia | F | CGCAGTTTGAAGATCAGCAG | 474668 | 474687 | 20 | 5594 |
|  |  | TPI-33AR | R | GCCATCACGTATGTGCGATGGC | 480240 | 480261 | 22 |  |
| POOL-2 | TPI-33B (FR1) | 33B-F | F | TTCTACCGATGTGCGTTCTG | 478327 | 478346 | 20 | 6489 |
|  |  | 33B-R1 | R | ATACGGGAAAGGACACGTTG | 484796 | 484815 | 20 |  |
| POOL-2 | TPI-33C (F2R2) | 33C-F2 | F | GAGATTTTTGCCGTGCATTC | 484363 | 484382 | 20 | 8527 |
|  |  | 33C-R2 | R | GCTGCCGTATTCTGTTCACC | 492870 | 492889 | 20 |  |
| POOL-2 | TPI-34A | 34A-F | F | CGGCTTCAATTTTTCTCAGC | 492652 | 492671 | 20 | 3702 |
|  |  | 34A-R | R | CTGATGACGACCAAACAACG | 496334 | 496353 | 20 |  |
| POOL-2 | TPI-34 (F3R3) | 34-F3 | F | ACCGCTACAAAGAGGATAGG | 495206 | 495225 | 20 | 4751 |
|  |  | 34-R3 | R | TCTACGCACAAAGAAAGAGC | 499937 | 499956 | 20 |  |
| POOL-2 | TPI-34rep (F4R5) | 34rep-F4 | F | GTCTTGTGCACATTATTCAAG | 497142 | 497162 | 21 | 772 |
|  |  | 34rep-R5 | R | CTTCGTGCAACATCGCTACG | 497894 | 497913 | 20 |  |
| POOL-2 | TPI-34B (F2R1) | 34B-F2 | F | GTTGCTTCTTCGGTCTGGTC | 498833 | 498852 | 20 | 4730 |
|  |  | 34B-R1 | R | AAGGATTTGCGTGGAACTTG | 503543 | 503562 | 20 |  |
| POOL-2 | TPI-36A | TPI-36-AF-Bosnia | F | TCGAGCAGCAGGGTAATCTC | 501154 | 501173 | 20 | 3719 |
|  |  | TPI-36-AR-Bosnia | R | CTACGCAGCGAAAACAGTGA | 504853 | 504872 | 20 |  |
| POOL-2 | TPI-36B | TPI-36-BF-Bosnia | F | CGTGGTCTCAAGCGGTAGTT | 504398 | 504417 | 20 | 4015 |
|  |  | TPI-36-BR-Bosnia | R | GCTTGCTATGCACCACTTGA | 508393 | 508412 | 20 |  |
|  |  |  |  |  |  |  |  |  |
| POOL-3 | TPI-37 | ES-167R | F | CCTGCAGGGTACGTAAGTAGAGGAC | 507390 | 507414 | 25 | 7040 |
|  |  | ES-146R | R | CGCTTTGATGAGGGAATAGAAGAC | 514406 | 514429 | 24 |  |
| POOL-3 | TPI-38A-1 | ES-97F | F | TGACCCAATGAAATGACCCTTC | 513763 | 513784 | 22 | 2827 |
|  |  | TPI-38A-1R | R | TCGCCATAGTCCTAGAAACG | 516570 | 516589 | 20 |  |
| POOL-3 | TPI-38A-2_new | 38A-2_newF | F | GCAATGAACCAGACAAGCAA | 516466 | 516485 | 20 | 3779 |
|  |  | 38A-2_newR | R | CGCACTGACACGCTAAACAG | 520225 | 520244 | 20 |  |
| POOL-3 | TPI-38A-3 | TPI-38A-3F | F | CGGAGTAAACGGTGTTTCGT | 519894 | 519913 | 20 | 4345 |
|  |  | TPI-38AF | R | GGATCAAAGGCAGGATTGAA | 524219 | 524238 | 20 |  |
| POOL-3 | TPI-38B-A | TPI-38BR | F | CCACCTCCTGTTTACGCATT | 524167 | 524186 | 20 | 4487 |
|  |  | TPI-38B-1R | R | AACAGGAAAGCAACTTGTCG | 528634 | 528653 | 20 |  |
| POOL-3 | TPI-38B-B | TPI-38B-2F | F | ATGGCGTTGAGACAGAAGAG | 527994 | 528013 | 20 | 3984 |
|  |  | TPI38B-2R | R | ACAATCCCTCTTCCTTCCAC | 531958 | 531977 | 20 |  |
| POOL-3 | TPI-38B-C | TPI-38B-3F | F | CGCTGGCAGAGTGTTATGAA | 531545 | 531564 | 20 | 4675 |
|  |  | ES-100R | R | CAGCATGGTCAGAAGCAATAGGTAG | 536195 | 536219 | 25 |  |
| POOL-3 | TPI-39A-A | ES-106F | F | GGCAGGGTTACATGCTTGTCTC | 535637 | 535658 | 22 | 4356 |
|  |  | 39A-A_newR | R | GGACTGCATTGGCTTTTGTT | 539973 | 539992 | 20 |  |
| POOL-3 | TPI-39A-B | 39A-B_newF | F | ACTGAACGAACACCCCTCTG | 539749 | 539768 | 20 | 4113 |
|  |  | 39A-B_newR | R | ACATCTGCTCCAAAGCCAAC | 543842 | 543861 | 20 |  |
| POOL-3 | TPI-39A-C | 39A-C_newF | F | GTGGCAGGTAGGTCTTTGGA | 543335 | 543354 | 20 | 4269 |
|  |  | TPI-39AR | R | CGACACAACTGGGTTTTCCT | 547584 | 547603 | 20 |  |
| POOL-3 | TPI-39B-A | TPI-39B-AF-Bosnia | F | TTGGAGTGTTGTTGCCAGAC | 544719 | 544738 | 20 | 6324 |
|  |  | TPI-39B-AR-Bosnia | R | GAAATGCGCTCGTGTAAGTG | 551023 | 551042 | 20 |  |
| POOL-3 | TPI-39B-B | TPI-39B-BF-Bosnia | F | AGAAGGACAGCGTCGGTATG | 550611 | 550630 | 20 | 6140 |
|  |  | TPI-39B-BR-Bosnia | R | TTTTGCCCCTATGATTGCAG | 556731 | 556750 | 20 |  |
| POOL-3 | TPI-40A | TPI-40-AF-Bosnia | F | CGAACCTACTACCGGATTGC | 553077 | 553096 | 20 | 5961 |
|  |  | TPI-40-AR-Bosnia | R | GCCCGATTTTGTTATTGGAG | 559018 | 559037 | 20 |  |
| POOL-3 | TPI-40B | TPI-40-BF-Bosnia | F | CCAAAGCAATATCCCAGTCC | 558762 | 558781 | 20 | 5239 |
|  |  | TPI-40-BR-Bosnia | R | GCGCGTGTTCTTTCATGTAG | 563981 | 564000 | 20 |  |
| POOL-3 | TPI-40C | TPI-40-CF-Bosnia | F | ATCTTGAGCACGAAGGATGC | 563653 | 563672 | 20 | 6451 |
|  |  | TPI-40-CR-Bosnia | R | CTTTGCGTAGGGTGCTCTTC | 570084 | 570103 | 20 |  |
| POOL-3 | TPI-40D | TPI-40-DF-Bosnia | F | CAGCCCGCTAATCTTTTCAG | 569532 | 569551 | 20 | 4959 |
|  |  | TPI-40-DR-Bosnia | R | GGTCCTTGCTCGTCTACTCG | 574471 | 574490 | 20 |  |
| POOL-3 | TPI-41A-A | ES-82R | F | TCACCACCTTTGACAGTACCCC | 571737 | 571758 | 22 | 4663 |
|  |  | 41A-A_newR | R | CGACTCCTTTCGTTGCTTTC | 576380 | 576399 | 20 |  |
| POOL-3 | TPI-41A-B | 41A-B_newF | F | TTGGAGAAAAATCCCGACAC | 575716 | 575735 | 20 | 4635 |
|  |  | TPI-41AR | R | GATCACGTATGTGCGTCCAC | 580331 | 580350 | 20 |  |
| POOL-3 | TPI-41B-A | TPI-41B-AF-Bosnia | F | CACACAGGCTCATACGCAAC | 579299 | 579318 | 20 | 3808 |
|  |  | TPI-41B-AR-Bosnia | R | GCTGCGGTCTACCTTCACTC | 583087 | 583106 | 20 |  |
| POOL-3 | TPI-41B-B | TPI-41B-BF-Bosnia | F | TGGATGTCCTTCACAATTCG | 582574 | 582593 | 20 | 5425 |
|  |  | TPI-41B-BR-Bosnia | R | ACAGTCGGGTGAAAGGACAG | 587979 | 587998 | 20 |  |
| POOL-3 | TPI-41B-C | TPI-41B-CF-Bosnia | F | TTCTGCCGTACCGAATACTG | 587619 | 587638 | 20 | 5563 |
|  |  | TPI-41B-CR-Bosnia | R | ATGTGCTCTGGCTGAAGGAG | 593162 | 593181 | 20 |  |
| POOL-3 | TPI-42A-A | TPI-42AF | F | GTTCTGCCATGCGCACCGCCATACG | 591445 | 591469 | 25 | 5431 |
|  |  | 42A-A_newR | R | GGATTTTGGACGGAAAGACA | 596856 | 596875 | 20 |  |
| POOL-3 | TPI-42A-B | 42A-B_newF | F | ATGATTTACCCGCATTTGGA | 596342 | 596361 | 20 | 5230 |
|  |  | TPI-42AR | R | TGGCGGCTACCTTCGCAGAACTTGC | 601547 | 601571 | 25 |  |
| POOL-3 | TPI-42B-A | TPI-42BF | F | TGCAAAGCAAATATCGCAATGAGC | 601025 | 601048 | 24 | 5529 |
|  |  | 42B-A_newR | R | TGAGGCAGGGGTGTATCAAG | 606534 | 606553 | 20 |  |
| POOL-3 | TPI-42B-B | 42B-B_newF | F | ACCAAAACACCCAGTGCTTC | 606191 | 606210 | 20 | 6038 |
|  |  | TPI-42BR2 | R | ACATCATTGCAAGCATTATCCG | 612207 | 612228 | 22 |  |
| POOL-3 | TPI-43A | ES-88R | F | GGATGGAAAATTCGTTGTACCCT | 611593 | 611615 | 23 | 5578 |
|  |  | TPI-43AF | R | GCGTTTTCCTGTTGATTGGT | 617151 | 617170 | 20 |  |
| POOL-3 | TPI-43D | TPI-43DF | F | ACGGTACAACGGAGAAATCG | 616200 | 616219 | 20 | 3993 |
|  |  | TPI-43DR | R | CTGCTCGGTTAGGACTGGAC | 620173 | 620192 | 20 |  |
| POOL-3 | TPI-43B | TPI-43BR | F | TACCGTCCATCTGTCCACAA | 619214 | 619233 | 20 | 5301 |
|  |  | TPI-43BF | R | AGTTTTTCCCCCACTGCTTT | 624495 | 624514 | 20 |  |
| POOL-3 | TPI-43C | TPI-43CR | F | ATGCCCTCTCTGCTACCTCA | 624061 | 624080 | 20 | 7841 |
|  |  | ES-89F | R | CAAGCGCAGTAAAATCCCTCAG | 631880 | 631901 | 22 |  |
| POOL-3 | TPI-44A-A | ES-143R | F | GCGTTTTTCCTGTTCTCCCTCT | 631372 | 631393 | 22 | 4908 |
|  |  | 44A-A_newR | R | TCGTGCGGGAAATAAATAGC | 636260 | 636279 | 20 |  |
| POOL-3 | TPI-44A-B | 44A-B_newF2 | F | CAACAAATGCAAGCAGATCG | 635944 | 635963 | 20 | 2585 |
|  |  | 44A-B_newR | R | ACTGGGGCATTGTGTTTGTT | 638509 | 638528 | 20 |  |
| POOL-3 | TPI-44B-A | TPI-44B-AF-Bosnia | F | GAGAACCCATCCCTCTTGTG | 638322 | 638341 | 20 | 3294 |
|  |  | TPI-44B-AR-Bosnia | R | CGTCTCATGCGTGTTCTCTC | 641596 | 641615 | 20 |  |
| POOL-3 | TPI-44B-B | TPI-44B-BF-Bosnia | F | GATAGAACCGACCCCGTACA | 641316 | 641335 | 20 | 6839 |
|  |  | TPI-44B-BR-Bosnia | R | CATTGAAGTGCTCGGTGATG | 648135 | 648154 | 20 |  |
| POOL-3 | TPI-44B-C | TPI-44B-CF-Bosnia | F | AGCCTGATCTAGCACCCTCA | 647880 | 647899 | 20 | 4232 |
|  |  | TPI-44B-CR-Bosnia | R | ATTGACTGTGCTGCGTTCAG | 652092 | 652111 | 20 |  |
| POOL-3 | TPI-45A | TPI-45-AF-Bosnia | F | AGGATCGGAATGGGGAGTAG | 651728 | 651747 | 20 | 4223 |
|  |  | TPI-45-AR-Bosnia | R | TTGGGATAAAACGCTCCTTG | 655931 | 655950 | 20 |  |
| POOL-3 | TPI-45B | TPI-45-BF-Bosnia | F | GCCTCTTCTGCTGCTTTCAG | 655681 | 655700 | 20 | 4771 |
|  |  | TPI-45-BR-Bosnia | R | TAGGGCCGAAGGTGTAGATG | 660432 | 660451 | 20 |  |
| POOL-3 | TPI-46A | 46A_newF | F | ACACCGATCATTACCGCTTC | 660217 | 660236 | 20 | 3100 |
|  |  | 46A_newR | R | TAGTCATTCCGCACACGAAG | 663297 | 663316 | 20 |  |
| POOL-3 | TPI-46B | 46B_newF | F | TGTGGAAACTCCCTGACACC | 662987 | 663006 | 20 | 3595 |
|  |  | 46B_newR | R | CGTCTTTTACCACCCCACAG | 666562 | 666581 | 20 |  |
| POOL-3 | TPI-47 | ES-171F | F | GCATAACCGCGTGCTATACGA | 666106 | 666126 | 21 | 2003 |
|  |  | ES-172R | R | AGCGCCTCTACATCCTCCATG | 668088 | 668108 | 21 |  |
| POOL-3 | TPI-48-1 | 48-1F | F | ACTGTGTGCAGCGATTGAGTATTG | 667779 | 667802 | 24 | 3928 |
|  |  | 48-1R | R | CATGCTGCTAACCTGGCTTGAG | 671685 | 671706 | 22 |  |
| POOL-3 | TPI-48-2 | 48-2F | F | GCACGGATGTATGCGGTGTAAG | 671288 | 671309 | 22 | 4017 |
|  |  | 48-2R | R | CTGGATACCTTGATTGGGCAGTAG | 675281 | 675304 | 24 |  |
| POOL-3 | TPI-48-3 | 48-3F | F | TCCCTGGAAGAGGTTATTTGAGAGG | 674271 | 674295 | 25 | 4247 |
|  |  | 48-3R | R | ACTGCATGCTCTTTCTGATCCATTAC | 678492 | 678517 | 26 |  |
| POOL-3 | TPI-49A | ES-34R | F | GCCACTCACCTGAAGATTGGAC | 678390 | 678411 | 22 | 5018 |
|  |  | 49A_newR | R | GTACCCGCTTCTCTTTGCAG | 683388 | 683407 | 20 |  |
| POOL-3 | TPI-49B | 49A_newF | F | AGTGCGAACAAGATGCACAG | 683086 | 683105 | 20 | 5520 |
|  |  | ES-31F | R | TCGAAGCGAACCATTTTCCTAAT | 688583 | 688605 | 23 |  |
| POOL-3 | TPI-50A-A | ES-68R | F | TGGTTCGCTTCGATTATCATAACTTAA | 688593 | 688619 | 27 | 4554 |
|  |  | 50A-A_newR | R | ATCGGAGTGCAACAACTTCC | 693127 | 693146 | 20 |  |
| POOL-3 | TPI-50A-B | 50A-B_newF | F | AGGCATTTCTCGTCCAACAC | 692658 | 692677 | 20 | 4355 |
|  |  | 50A-B_newR | R | ATGGTTCTGGTGGATGTGGT | 696993 | 697012 | 20 |  |
| POOL-3 | TPI-50A-C_new | 50A-C_newF2 | F | GTGCTGCCTCACAAAGATGA | 696878 | 696897 | 20 | 1746 |
|  |  | 50A-C_newR2 | R | AGGTGCGGGTTTCTCTAGGT | 698604 | 698623 | 20 |  |
| POOL-3 | TPI-50B-A | TPI-50B-AF-Bosnia | F | TCTGCACCACTCCTTGTACG | 698401 | 698420 | 20 | 5561 |
|  |  | TPI-50B-AR-Bosnia | R | GTCGCCATAAGAACCTCAGC | 703942 | 703961 | 20 |  |
| POOL-3 | TPI-50B-B | TPI-50B-BF-Bosnia | F | CGAGTGTCGTGAACCTTGAG | 703604 | 703623 | 20 | 5643 |
|  |  | TPI-50B-BR-Bosnia | R | TGTGCCATTTCTCACTCCTG | 709227 | 709246 | 20 |  |
| POOL-3 | TPI-52A-A | ES-67R | F | AGTCATTGCGTTTTTCTCGGG | 708207 | 708227 | 21 | 4512 |
|  |  | 52A-A_newR | R | TCAAGAGATGTTGCGTTTGC | 712699 | 712718 | 20 |  |
| POOL-3 | TPI-52A-B1 | SDTP0648-0649L | F | CAAAGCGAGCATCTACGTCA | 711454 | 711473 | 20 | 4270 |
|  |  | SDTP0651-0652R | R | GGTCTGTCGCACTCCTTCTC | 715704 | 715723 | 20 |  |
| POOL-3 | TPI-52A-B3 | SDTP0651-0652L | F | AACGTGTCATCGTCAAGCTG | 715022 | 715041 | 20 | 5227 |
|  |  | SDTP0655-0658R | R | GCAGTGCAGGTTCCGTTTAT | 720229 | 720248 | 20 |  |
| POOL-3 | TPI-52B | TP-52BF | F | ACCGTTTTCCAGTCGTATGC | 715964 | 715983 | 20 | 7399 |
|  |  | ES-170R | R | GGTCAGATAGGACAAGGGGTCAC | 723340 | 723362 | 23 |  |
| POOL-3 | TPI-53A | ES-23R | F | CATGACCGTTTCAAAGGGCTC | 721173 | 721193 | 21 | 6093 |
|  |  | TP-53AR | R | GCAACCTCTCCTGTCAAAGC | 727246 | 727265 | 20 |  |
| POOL-3 | TPI-53B-A_new | 53B-A_F_Pa | F | GATGACATGTCTGATGCCTGGT | 726975 | 726996 | 22 | 5001 |
|  |  | 53B-A_R_Pa | R | TTTGCTGAGCGTGCAGAGAAAC | 731954 | 731975 | 22 |  |
| POOL-3 | TPI-53B-B | 53B-B_newF | F | TGCAGTTTGATAGGCAACCA | 731838 | 731857 | 20 | 5678 |
|  |  | ES-22F | R | CAGTTTGCTCTACCTGCGTCCT | 737494 | 737515 | 22 |  |
| POOL-3 | TPI-54A-A1 | TPI-54A-AF-Bosnia | F | GTAATGAGATTCGCCGGAAC | 736349 | 736368 | 20 | 4530 |
|  |  | 54A-A1_newR | R | AAGGGAAGAGAGCGTTGGAG | 740859 | 740878 | 20 |  |
| POOL-3 | TPI-54A-A2 | 54A-A2_newF | F | GAGGCGTACCTTCTCCATCC | 740120 | 740139 | 20 | 4239 |
|  |  | TPI-54A-AR_new | R | AAAGGGCATCGGTATGATGT | 744339 | 744358 | 20 |  |
| POOL-3 | TPI-54A-B | TPI-54A-BF-Bosnia | F | ATGCTTCCAGTCCGCTACAG | 744107 | 744126 | 20 | 6754 |
|  |  | TPI-54A-BR-Bosnia | R | GGCTCATCAAGCAAGAGGAC | 750841 | 750860 | 20 |  |
| POOL-3 | TPI-54B-A | TPI-54B-AF-Bosnia | F | CCGAGGAACGTAAGCTCAAC | 750596 | 750615 | 20 | 6095 |
|  |  | TPI-54B-AR-Bosnia | R | ATTATCTCATGCGCCGTACC | 756671 | 756690 | 20 |  |
| POOL-3 | TPI-54B-B | TPI-54B-BF-Bosnia | F | AAAGCAGTCCGTACATCACG | 756318 | 756337 | 20 | 5606 |
|  |  | TPI-54B-BR-Bosnia | R | ATACGCGAGGAAGAGCTACG | 761904 | 761923 | 20 |  |
|  |  |  |  |  |  |  |  |  |
| POOL-4 | TPI-55A | TPI-55-AF-Bosnia | F | TCGATAAAGGCTGCGATACC | 759633 | 759652 | 20 | 4843 |
|  |  | TPI-55-AR-Bosnia | R | AGCACTCTCGCATTGTGTTG | 764456 | 764475 | 20 |  |
| POOL-4 | TPI-55B | TPI-55-BF-Bosnia | F | ACCAGGCACCACGCTACTAC | 764373 | 764392 | 20 | 4617 |
|  |  | TPI-55-BR-Bosnia | R | CAGCTATTGCACCGTATGGA | 768970 | 768989 | 20 |  |
| POOL-4 | TPI-55C | TPI-55-CF-Bosnia | F | AAACCGCGTCAACTACATCC | 768647 | 768666 | 20 | 5604 |
|  |  | TPI-55-CR-Bosnia | R | GAGAAAAGCGGGAGGTAGTG | 774231 | 774250 | 20 |  |
| POOL-4 | TPI-56A | ES-15R | F | CCATCGTTACCGTTTTCTCTAGCA | 771591 | 771614 | 24 | 7720 |
|  |  | TPI-56AF | R | TTCGCAGGATATTTGGAAGG | 779291 | 779310 | 20 |  |
| POOL-4 | TPI-56D | TPI-56DF | F | CAGATAAGGAGGCGCTTGAG | 778345 | 778364 | 20 | 2391 |
|  |  | TPI-56DR | R | AACTGGTATTCAGCCACGAG | 780716 | 780735 | 20 |  |
| POOL-4 | TPI-56B | TPI-56BR | F | CGTTGCAATATTCGTCTTTCC | 780020 | 780040 | 21 | 1017 |
|  |  | TPI-56BF | R | TAGCACAGGAGTTGCAGTCG | 781017 | 781036 | 20 |  |
| POOL-4 | TPI-56C | TPI-56CR | F | ACCGCACGAAAGTATTCCAC | 780490 | 780509 | 20 | 8562 |
|  |  | ES-14F | R | TGAAAATTCAGTAATGGAAGGGGTC | 789027 | 789051 | 25 |  |
| POOL-4 | TPI-57A-A | TPI-57A-AF-Bosnia | F | AAACATGCACCGAAAGGAAC | 787365 | 787384 | 20 | 7043 |
|  |  | TPI-57A-AR-Bosnia | R | CCGAGGATTTGCTACCACTC | 794388 | 794407 | 20 |  |
| POOL-4 | TPI-57A-B | TPI-57A-BF-Bosnia | F | TTATTCTCCGACAGGGCAAG | 794231 | 794250 | 20 | 6465 |
|  |  | TPI-57A-BR-Bosnia | R | CCTGCCGAAAGAAGTGGTAG | 800676 | 800695 | 20 |  |
| POOL-4 | TPI-57B/58-1 | TPI-57BF | F | TGCCCATTTCATCAGCAACC | 799350 | 799369 | 20 | 4611 |
|  |  | 57B/58-1_newR | R | GGTGCTGGACTTTGGGTCTA | 803941 | 803960 | 20 |  |
| POOL-4 | TPI-57B/58-2 | 57B/58-2_newF | F | GGAAGTTTGGAAGTGCAAGG | 803429 | 803448 | 20 | 5427 |
|  |  | 57B/58-2_newR | R | CGTGAGGTGTTGATGATTGG | 808836 | 808855 | 20 |  |
| POOL-4 | TPI-57B/58-3 | 57B/58-3_newF | F | TCCGCAGTGAAACACAAGAG | 808395 | 808414 | 20 | 4780 |
|  |  | 57B/58-3_newR | R | GCCTTTGCTTCGTTTAGTCG | 813155 | 813174 | 20 |  |
| POOL-4 | TPI-57B/58-4 | 57B/58-4_newF | F | TCGATGAGTAGCACCGACAG | 812617 | 812636 | 20 | 4989 |
|  |  | 57B/58-4_newR | R | GCTCTCGCTCTTTGCTCAGT | 817586 | 817605 | 20 |  |
| POOL-4 | TPI-57B/58-5 | 57B/58-5_newF | F | GCTTTGCGGATACCTCTCTG | 817182 | 817201 | 20 | 5025 |
|  |  | ES-8F | R | CGCTGCACTGTACCACAAGTAGAC | 822183 | 822206 | 24 |  |
| POOL-4 | TPI-59A | TPI-59-AF-Bosnia | F | AGAACTCCATCGCCTGTCTG | 820430 | 820449 | 20 | 4352 |
|  |  | TPI-59-AR-Bosnia | R | TCCTGGGCTGGTAAGAAATG | 824762 | 824781 | 20 |  |
| POOL-4 | TPI-59B | TPI-59-BF-Bosnia | F | TGACACTTCCAAACGAGCAG | 824343 | 824362 | 20 | 5794 |
|  |  | TPI-59-BR-Bosnia | R | TGTCGGTTCCCCTTACTTTG | 830117 | 830136 | 20 |  |
| POOL-4 | TPI-59C-2 | TPI-59-CF-Bosnia-new | F | CCTGAGCGTAAAGGAACAGC | 828342 | 828361 | 20 | 2870 |
|  |  | TPI-59-CR-Bosnia-new | R | TGCAACTGAAAGGCACAGAG | 831192 | 831211 | 20 |  |
| POOL-4 | TPI-59D-A | TPI-59-DF-Bosnia-new | F | GAAAGACACGGCACTCTTCC | 831129 | 831148 | 20 | 3704 |
|  |  | TPI-59D-inner-R-Bosnia | R | TGCGTTGTGTTAAGCCTGTC | 834813 | 834832 | 20 |  |
| POOL-4 | TPI-59D-B | TPI-59D-inner-F-Bosnia | F | CGGAAGAATCGTGGAAGAAG | 834328 | 834347 | 20 | 2805 |
|  |  | TPI-59-DR-Bosnia-new | R | AGCGATACCCTGTGATCCTG | 837113 | 837132 | 20 |  |
| POOL-4 | TPI-60A | TPI-60-AF-Bosnia | F | CCTTCATGGGAAGCTACGAG | 836696 | 836715 | 20 | 5954 |
|  |  | TPI-60-AR-Bosnia | R | ATGGAATGCTTGCGAAGAAG | 842630 | 842649 | 20 |  |
| POOL-4 | TPI-60B | TPI-60-BF-Bosnia | F | GACTTGCTATCGGGTTGGAG | 842190 | 842209 | 20 | 5827 |
|  |  | TPI-60-BR-Bosnia | R | CTAGCACTGGTTGTGCAGGA | 847997 | 848016 | 20 |  |
| POOL-4 | TPI-60C | TPI-60-CF-Bosnia | F | ACACCGCATCAGCACTAGAC | 847692 | 847711 | 20 | 4981 |
|  |  | TPI-60-CR-Bosnia | R | GTACGTTGTGGTCCGTGATG | 852653 | 852672 | 20 |  |
| POOL-4 | TPI-60D | TPI-60-DF-Bosnia | F | AATGGAGCAGGCAAATCAGT | 852192 | 852211 | 20 | 5745 |
|  |  | TPI-60-DR-Bosnia | R | GCAAATCGGAGGTAGAATCC | 857917 | 857936 | 20 |  |
| POOL-4 | TPI-61A | TPI-61-AF-Bosnia | F | CTCCGTGGAAAGCTAAGACG | 857744 | 857763 | 20 | 6818 |
|  |  | TPI-61-AR-Bosnia | R | TTTCTGAGCGCGTTGTACTG | 864542 | 864561 | 20 |  |
| POOL-4 | TPI-61B-2A | TPI-61B-BF-Bosnia-new | F | TATGCAGCGCAAGACATACC | 863716 | 863735 | 20 | 4245 |
|  |  | TPI-61B-inner-R-Bosnia | R | CTGATAGCCGTGCGTACATC | 867941 | 867960 | 20 |  |
| POOL-4 | TPI-61B-2B | TPI-61B-inner-F-Bosnia | F | ACTCGCAGGTATGAACATCG | 867373 | 867392 | 20 | 3538 |
|  |  | TPI-61B-BR-Bosnia-new | R | TGCGTATATGAACGGACTGC | 870891 | 870910 | 20 |  |
| POOL-4 | TPI-61C | TPI-61-CF-Bosnia | F | GTCTTTCCCACACCAGCTTC | 870009 | 870028 | 20 | 5200 |
|  |  | TPI-61-CR-Bosnia | R | AGCAGTTTACGCCCTTTCTG | 875189 | 875208 | 20 |  |
| POOL-4 | TPI-62A-A1 | TPI-62A-AF-Bosnia-new | F | AATCTGTGCGTGCTTGAGTG | 874933 | 874952 | 20 | 4387 |
|  |  | TPI-62A-AR-Bosnia-new | R | ACTGAAGGTGCCAAGGTACG | 879300 | 879319 | 20 |  |
| POOL-4 | TPI-62A-A2 | TPI-62A-BF-Bosnia-new | F | CGTTCGTGACCGTGTAGAAG | 878884 | 878903 | 20 | 4678 |
|  |  | TPI-62A-BR-Bosnia-new | R | GCACCATCCGAAGTAGCAGT | 883542 | 883561 | 20 |  |
| POOL-4 | TPI-62A-B | TPI-62A-BF-Bosnia | F | TGATTGAGAGCAAGGCACAC | 882781 | 882800 | 20 | 6565 |
|  |  | TPI-62A-BR-Bosnia | R | ACGTATCGGCATGTGAGTGA | 889326 | 889345 | 20 |  |
| POOL-4 | TPI-62B-A | TPI-62B-AF-Bosnia | F | CCCTTGTAGCGTTCCTTCAG | 888915 | 888934 | 20 | 6975 |
|  |  | TPI-62B-AR-Bosnia | R | ACCAGCAATGGTACGACCTC | 895870 | 895889 | 20 |  |
| POOL-4 | TPI-62B-B | TPI-62B-BF-Bosnia | F | GGAGGCACGCATATACAGGT | 895666 | 895685 | 20 | 5850 |
|  |  | TPI-62B-BR-Bosnia | R | TGACCTTTCCGCTCATTACC | 901496 | 901515 | 20 |  |
| POOL-4 | TPI-63A | TPI-63-AF-Bosnia | F | ATGCACACTTATCGCCACAC | 901189 | 901208 | 20 | 8392 |
|  |  | TPI-63-AR-Bosnia | R | ATGGAGTCATTGGAGCGAAC | 909561 | 909580 | 20 |  |
| POOL-4 | TPI-63B | TPI-63-BF-Bosnia | F | TCATCGAGCAAAAGCACATC | 909127 | 909146 | 20 | 8074 |
|  |  | TPI-63-BR-Bosnia | R | TCGAACTCATGCTCGTTCAC | 917181 | 917200 | 20 |  |
| POOL-4 | TPI-64A_new | 64A_newF | F | GCGCTACTTTGCCTATCAGC | 916874 | 916893 | 20 | 3052 |
|  |  | 64A_newR | R | CGATGAAGGGGATAAGGTGA | 919906 | 919925 | 20 |  |
| POOL-4 | TPI-64B | TPI-64-BF-Bosnia | F | TGGACATAGACCGACCTTCC | 919768 | 919787 | 20 | 5003 |
|  |  | TPI-64-BR-Bosnia | R | AATAAGCACGGAGTGGGAAC | 924751 | 924770 | 20 |  |
| POOL-4 | TPI-65A-A | 65A-F2 | F | CAATGGGAAGACCAGAGGAG | 924113 | 924132 | 20 | 4545 |
|  |  | 65A-A_newR | R | ACCGTCTCATCACTCCCAAC | 928638 | 928657 | 20 |  |
| POOL-4 | TPI-65A-B | 65A-B_newF | F | CGAATCCCGAGGAAATACAA | 928308 | 928327 | 20 | 4655 |
|  |  | 65A-R1 | R | AGAATCCATCAACGCTCTGC | 932943 | 932962 | 20 |  |
| POOL-4 | TPI-65B | TPI-65B-F2-Bosnia | F | GTCGGCAAGGTACTCATCGT | 932474 | 932493 | 20 | 5324 |
|  |  | TPI-65B-R-Bosnia | R | TGCTTGAGTGTGAGGCAGAC | 937778 | 937797 | 20 |  |
| POOL-4 | TPI-66A-A | TPI-66A-AF-Bosnia | F | ATGTCAGTCCCCTTGAGCAG | 937317 | 937336 | 20 | 4934 |
|  |  | TPI-66A-AR-Bosnia | R | CATGCACACGGTACAAAAGG | 942231 | 942250 | 20 |  |
| POOL-4 | TPI-66A-B | TPI-66A-BF-Bosnia | F | TTTGCCTGAAAAGGGATCTG | 942001 | 942020 | 20 | 4803 |
|  |  | TPI-66A-BR-Bosnia | R | TTGGATATGAAGGCGAGGTC | 946784 | 946803 | 20 |  |
| POOL-4 | TPI-66A-C | TPI-66A-CF-Bosnia | F | TCTGCCTCATTTTCCTTTGG | 946356 | 946375 | 20 | 5673 |
|  |  | TPI-66A-CR-Bosnia | R | AAATCAGCGATGTCCGAGAG | 952009 | 952028 | 20 |  |
| POOL-4 | TPI-66B-1 | 66B-1_F_Pa | F | TGGATACCGACGTCACCCTTAATC | 951803 | 951826 | 24 | 2009 |
|  |  | 66B-1_R_Pa | R | TCAAGTCCTGAGCCGAGAATG | 953791 | 953811 | 21 |  |
| POOL-4 | TPI-66B-2 | 66B-2_newF | F | ATGGAAAGCCAGGACAACAC | 953488 | 953507 | 20 | 4681 |
|  |  | 66B-2_newR | R | TGCTGTGGTGTTTTCTACGC | 958149 | 958168 | 20 |  |
| POOL-4 | TPI-66B-3 | 66B-3_F_Pa | F | CGCTGCTATTTGCGCTCTATTTCC | 958054 | 958077 | 24 | 4202 |
|  |  | 66B-3_R_Pa | R | GGCTGCGTAACAACCTTGAACTG | 962233 | 962255 | 23 |  |
| POOL-4 | TPI-67A-A | ES-130F | F | TGTGTGAGATTCAAATCCCAAGG | 962044 | 962066 | 23 | 4797 |
|  |  | 67A-A_newR | R | GTTGTTCGGCAGGCTTCTAC | 966821 | 966840 | 20 |  |
| POOL-4 | TPI-67A-B | 67A-B_newF | F | TAATCTCACGCACCGTCAAG | 966559 | 966578 | 20 | 5165 |
|  |  | TPI-67-R1 | R | GTCTCGGAAGTGGATTTGTC | 971704 | 971723 | 20 |  |
| POOL-4 | TPI-67B-A | 67B-F | F | GCAGGCTTACCAGCACCTAC | 969687 | 969706 | 20 | 4229 |
|  |  | 67B-A_newR | R | AGCCCACAAACAGAACTTGG | 973896 | 973915 | 20 |  |
| POOL-4 | TPI-67B-B | 67B-B_newF | F | TGGTCGGATAGGAATTTTCG | 973468 | 973487 | 20 | 4410 |
|  |  | 67B-R | R | AGTTTCGGGTGATTGGAGTG | 977858 | 977877 | 20 |  |
| POOL-4 | TPI-67C-A | 67C-F | F | AGTACCCCGCTGACAATCAC | 976755 | 976774 | 20 | 4908 |
|  |  | 67C-A_newR | R | TATGTGGAGCGTGAGTTTCG | 981643 | 981662 | 20 |  |
| POOL-4 | TPI-67C-B | 67C-B_newF | F | TCTGCTTCCATGAACGAATG | 981419 | 981438 | 20 | 4178 |
|  |  | 67C-R | R | CAGCAGTGTATCGTGCGTTC | 985577 | 985596 | 20 |  |
| POOL-4 | TPI-68A-A | TPI-68A-AF-Bosnia | F | CATCTACCTTTCCGCTGCTC | 985152 | 985171 | 20 | 3224 |
|  |  | TPI-68A-AR-Bosnia | R | AGAGATTCATGGCGGATGAC | 988356 | 988375 | 20 |  |
| POOL-4 | TPI-68A-B | TPI-68A-BF-Bosnia | F | GCGACGGGATCATTCACTAC | 987970 | 987989 | 20 | 6732 |
|  |  | TPI-68A-BR-Bosnia | R | GTATCCACCACAGGGAGTGC | 994682 | 994701 | 20 |  |
| POOL-4 | TPI-68A-C_new | 68A-C_newF | F | GCTTTTCTCGCCACCATTAC | 994217 | 994236 | 20 | 2636 |
|  |  | 68A-C_newR | R | CGTGCATGTTCCGTAGAAGA | 996833 | 996852 | 20 |  |
| POOL-4 | TPI-68B-A | 68B-F | F | TCGCTTTCTACCTCCTCCAG | 996354 | 996373 | 20 | 4701 |
|  |  | 68B-A_newR | R | CGCGTCCGTAGTAGTCATCA | 1001035 | 1001054 | 20 |  |
| POOL-4 | TPI-68B-B | 68B-B_newF | F | GCGCAGTACGTGTTTCAGTC | 1000883 | 1000902 | 20 | 5214 |
|  |  | 68B-R | R | GCTTCTTCACTGGGATCGTC | 1006077 | 1006096 | 20 |  |
| POOL-4 | TPI-68C-A | 68C-F | F | GCTGCATCGTAGAGGGTTTG | 1005757 | 1005776 | 20 | 4491 |
|  |  | 68C-A_newR | R | TGAAGCCGTTTTGTGTCTTG | 1010228 | 1010247 | 20 |  |
| POOL-4 | TPI-68C-B | 68C-B_newF | F | TGCTAAACACGGCAAAGATG | 1010024 | 1010043 | 20 | 4001 |
|  |  | 68C-R | R | AGTACGTCCAGCCAATCCAG | 1014005 | 1014024 | 20 |  |
| POOL-4 | TPI69A-A | ES-134F | F | CCATCAGCACAAAGGGTGTGT | 1007905 | 1007925 | 21 | 4426 |
|  |  | 69A-A_newR | R | TGGGAAGCGTATCTGCCTAC | 1012311 | 1012330 | 20 |  |
| POOL-4 | TPI69A-B | 69A-B_newF | F | CGCAGACATAGAACGCATGT | 1011534 | 1011553 | 20 | 4558 |
|  |  | TPI-69AF | R | GTGCGTTGCACCCCTATACT | 1016072 | 1016091 | 20 |  |
| POOL-4 | TPI-69B | TPI-69BR | F | CTGGGGTGTTCACTGACCTT | 1015866 | 1015885 | 20 | 6792 |
|  |  | TPI-69BF | R | GTCAGGCGGTGTTCTATGGT | 1022638 | 1022657 | 20 |  |
| POOL-4 | TPI-69C | TPI-69CR | F | CTCAGCTTCCGGCAAGATAC | 1022179 | 1022198 | 20 | 4101 |
|  |  | TPI-69CF | R | GCTCACGAAGGAGGAGTACG | 1026260 | 1026279 | 20 |  |
| POOL-4 | TPI-69D | TPI-69DR | F | GCACACCCCAACTGCTTAAT | 1025521 | 1025540 | 20 | 7142 |
|  |  | ES-137R | R | CAGAGTGGACCCTCGTGTTCTTAG | 1032639 | 1032662 | 24 |  |
| POOL-4 | TPI-70A | ES-136F | F | CCATACACATCCATGTACTCGCAC | 1031892 | 1031915 | 24 | 7551 |
|  |  | TPI-70AR | R | AACGCGGTGCATCGGTCTGTCCAC | 1039419 | 1039442 | 24 |  |
| POOL-4 | TPI-70B | TPI-70BF | F | AAGCCGGGGTCTCCATCGTCCATCTT | 1038942 | 1038967 | 26 | 7252 |
|  |  | ES-157F | R | AGTATTTTGCGCGTAGGCGTC | 1046173 | 1046193 | 21 |  |
| POOL-4 | TPI-71A-3 | ES-158R | F | GCCATAGGAAACCGTAAGACCG | 1045435 | 1045456 | 22 | 4489 |
|  |  | TPI-71-R20 | R | CGGGATTTTATCATCTCTGC | 1049904 | 1049923 | 20 |  |
| POOL-4 | TPI-71A-7 | TPI-71-F15 | F | GTAAAGCGGGTGTCAAGACG | 1049691 | 1049710 | 20 | 801 |
|  |  | TPI-71-F11rc-Luis | R | CCAGTACGATAAGCAGCGG | 1050473 | 1050491 | 19 |  |
| POOL-4 | TPI-71A-6 | TPI-71A-F17 | F | GCTGCGTTACCGTCATACC | 1050325 | 1050343 | 19 | 3872 |
|  |  | TPI71-AR2 | R | GTTCTGGAGCGAGCCATTAG | 1054177 | 1054196 | 20 |  |
| POOL-4 | TPI-71B | TP-71BF | F | CAAGCGCCAGAAGATACTCC | 1052540 | 1052559 | 20 | 5178 |
|  |  | ES-139R | R | CGAGGATTTTGAGCATGCCTAG | 1057696 | 1057717 | 22 |  |
| POOL-4 | TPI-72A-A | ES-138F | F | CGTAAGTGATCGCATGTCCCTC | 1056837 | 1056858 | 22 | 4195 |
|  |  | 72A-A_newR | R | CCAGAGTCCTACGGCAGTTC | 1061012 | 1061031 | 20 |  |
| POOL-4 | TPI-72A-B | 72A-B_F | F | CTCCTGTGATGTCGCTCTCA | 1060680 | 1060699 | 20 | 4242 |
|  |  | 72A-B_R | R | AGATCGCCACACAGATGACC | 1064902 | 1064921 | 20 |  |
| POOL-4 | TPI-72B-A | TP-72BF | F | GCAACCTTCGCTCTTTGTTC | 1064583 | 1064602 | 20 | 4118 |
|  |  | 72B-A_newR | R | CACGGGTTCTTTTGTCTGGT | 1068681 | 1068700 | 20 |  |
| POOL-4 | TPI-72B-B | 72B-B_newF | F | GAGCCATGTATCCTCCCAGA | 1068552 | 1068571 | 20 | 4574 |
|  |  | ES-155F | R | CGTTTGAGCACGAAGGGTACC | 1073105 | 1073125 | 21 |  |
| POOL-4 | TPI-73A | TPI-73-AF-Bosnia | F | GCTGCTCATTCTTTCCATCC | 1072506 | 1072525 | 20 | 5532 |
|  |  | TPI-73-AR-Bosnia | R | ACGGCAAAAAGGTAATCGTG | 1078018 | 1078037 | 20 |  |
| POOL-4 | TPI-73B | TPI-73-BF-Bosnia | F | ACCGACTTCATCCCTGACAC | 1077491 | 1077510 | 20 | 5935 |
|  |  | TPI-73-BR-Bosnia | R | ACGATCAGCGACGAGGTTAG | 1083406 | 1083425 | 20 |  |
| POOL-4 | TPI-74A-A | TPI-74A-AF-Bosnia | F | TCTTTCGCTGCATGTGCTAC | 1082842 | 1082861 | 20 | 4093 |
|  |  | TPI-74A-AR-Bosnia | R | GATAGGAGAGAGGGCCATGA | 1086915 | 1086934 | 20 |  |
| POOL-4 | TPI-74A-B | TPI-74A-BF-Bosnia | F | TTGCCTTCCCACTCTTGTTC | 1086303 | 1086322 | 20 | 6851 |
|  |  | TPI-74A-BR-Bosnia | R | ACCGGCGATTGAAGAAGTAG | 1093134 | 1093153 | 20 |  |
| POOL-4 | TPI-74A-C | TPI-74A-CF-Bosnia | F | CATACAAGGCGGTGGTATCC | 1092699 | 1092718 | 20 | 3874 |
|  |  | TPI-74A-CR-Bosnia | R | TGGAGTTTCTGTGGTCAACG | 1096553 | 1096572 | 20 |  |
| POOL-4 | TPI-74B-A | TPI-74B-AF-Bosnia | F | CACTCCCCGCTTCTCTACTG | 1096449 | 1096468 | 20 | 4987 |
|  |  | TPI-74B-AR-Bosnia | R | TCAGAGGTTGCGTACTGCTG | 1101416 | 1101435 | 20 |  |
| POOL-4 | TPI-74B-B | TPI-74B-BF-Bosnia | F | CTACTTCGGGCAAACGACTC | 1100807 | 1100826 | 20 | 3599 |
|  |  | TPI-74B-BR-Bosnia | R | GGTGCTCCTCTCCATCAATC | 1104386 | 1104405 | 20 |  |
| POOL-4 | TPI-74B-C | TPI-74B-CF-Bosnia | F | CGTCATTGCTGTTGGTTCTG | 1104296 | 1104315 | 20 | 4129 |
|  |  | ES-126F | R | CACAGCGAGTATTTAAGGGTGGG | 1108402 | 1108424 | 23 |  |
| POOL-4 | TPI-75A | ES-127R | F | TTCTTGCCTTAATCTGTCCGAGC | 1107726 | 1107748 | 23 | 7310 |
|  |  | TPI-75AF | R | TGGTGCGTCTTTCTTCTCCT | 1115016 | 1115035 | 20 |  |
| POOL-4 | TPI-75B-A | TPI-75BR | F | ACAAAGGGCAAGAACACCAC | 1114098 | 1114117 | 20 | 4658 |
|  |  | TPI-75B-1R | R | CGATTTTCTTCTGCGAGGTC | 1118736 | 1118755 | 20 |  |
| POOL-4 | TPI-75B-B | TPI-75B-2F | F | GCCACACATGGACATACTCG | 1118378 | 1118397 | 20 | 3930 |
|  |  | ES-124F | R | CACCTATCTTTGTGTGGTAGGGGA | 1122284 | 1122307 | 24 |  |
| POOL-4 | TPI-76A | ES-125R | F | ATACCATTCTTCCAGCCCCGT | 1121473 | 1121493 | 21 | 1778 |
|  |  | ES-175R | R | CACCCTGACGGTGAGTCACTCA | 1123229 | 1123250 | 22 |  |
| POOL-4 | TPI-76B | 76B-F | F | GGTAAAGGTTACTGCCAGAATCTGCTC | 1122859 | 1122885 | 27 | 999 |
|  |  | 76B-R | R | ACCGCATTATGAGCGCACGAAAC | 1123835 | 1123857 | 23 |  |
| POOL-4 | TPI-77 (TP1030) | TP1030-XL-F2 | F | GTATGAGCGCATGGAGAAGG | 1123649 | 1123668 | 20 | 2384 |
|  |  | TP1030-XL-R2 | R | GAACGGCGTCTTTCTGTACG | 1126013 | 1126032 | 20 |  |
| POOL-4 | TPI-78 (F1R1) | TPI-78-F1-Bosnia | F | CAATCTGCTACGCGAAAAGG | 1125709 | 1125728 | 20 | 3006 |
|  |  | TPI-78-R1-Bosnia | R | GGACAGGGTTTGAGCTGTTC | 1128695 | 1128714 | 20 |  |
| POOL-4 | TPI-80 | ES-165R | F | CTCAATGTGCTGTTTGTAGTCGGA | 1128216 | 1128239 | 24 | 7949 |
|  |  | ES-153F | R | ACACTCCTCCTGCCTTGGAGAA | 1136143 | 1136164 | 22 |  |
|  |  |  |  |  |  |  |  |  |
| ^a^ TP intervals 11A and 25A were mixed to the Pool-4 | | |  |  |  |  |  |  |
| ^b^ primer coordinates, primer sequences, length of primers, and length of TP intervals according to the Nichols strain (GenBank AE000520.1) | | | | |  |  |  |  |
