# Supplementary material for: Nonhuman primates across sub-Saharan Africa are infected with the yaws bacterium Treponema pallidum subsp. pertenue
Source: Emerg Microbes Infect. 2018 Sep 19;7:157. doi: 10.1038/s41426-018-0156-4 (PMC6143531; doi:10.1038/s41426-018-0156-4)
Supplement: Supplementary file 7 — Supplementary Table S6 [file 41426_2018_156_MOESM7_ESM.docx]

**Table S6.** Summary of the PSGS sequencing results of four genomic DNA (gDNA) pools of the East African baboon genome (strain LMNP-1; MU, EKU, DPZ). Each gDNA pool resulted in overlapping PCR products. Processing was done on an Illumina Nextera NX library preparation and sequencing on MiSeq nano v2 2x250 bp. Raw reads in fastq format (past filter). *R1=forward read; R2=reverse read; Mean Qx%=percent bases with a Phred score of at least x; Mean Q=mean Phred score; Masked=reads smaller ten bases get masked with 35 consecutive "N". ** length according to the published *TPE* strain Fribourg-Blanc (Fribourg-Blanc) genome (17) (GenBank: NC_021179.1); nts=nucleotides.

| **Sample*** | **Pool** (nts)** | **No. of reads** | **No. of bases** | **Mean read length** | **Mean Q20%*** | **Mean Q30%*** | **Mean Q*** | **Masked*** |
| --- | --- | --- | --- | --- | --- | --- | --- | --- |
| M1_S19_L001  _R1_001 | 1  (259,918) | 216,572 | 38,354,135 | 178 | 97.43 | 96.32 | 37 | 1,153 |
| M1_S19_L001  _R2_001 |  | 216,572 | 38,449,943 | 178 | 93.89 | 91.58 | 36 | 1,151 |
| M2_S31_L001  _R1_001 | 2  (253,932) | 293,796 | 48,039,997 | 165 | 95.40 | 93.58 | 36 | 2,503 |
| M2_S31_L001  _R2_001 |  | 293,796 | 48,446,711 | 166 | 90.34 | 87.07 | 35 | 2,500 |
| M3_S43_L001  _R1_001 | 3  (254,562) | 313,125 | 52,076,572 | 167 | 97.24 | 96.09 | 37 | 2,138 |
| M3_S43_L001  _R2_001 |  | 313,125 | 52,293,004 | 168 | 93.04 | 90.60 | 36 | 2,123 |
| M4_S55_L001  _R1_001 | 4  (376,699) | 315,732 | 53,669,732 | 171 | 96.80 | 95.53 | 37 | 2,175 |
| M4_S55_L001  _R2_001 |  | 315,732 | 53,901,788 | 172 | 92.78 | 90.29 | 36 | 2,176 |
