# Supplementary material for: Nonhuman primates across sub-Saharan Africa are infected with the yaws bacterium Treponema pallidum subsp. pertenue
Source: Emerg Microbes Infect. 2018 Sep 19;7:157. doi: 10.1038/s41426-018-0156-4 (PMC6143531; doi:10.1038/s41426-018-0156-4)
Supplement: Supplementary file 8 — Supplementary Table S7 [file 41426_2018_156_MOESM8_ESM.docx]

**Table S7.** Number of nucleotide differences (i.e. indels and SNVs) of various lengths between the genome of the baboon (strain LMNP-1) and the published *TPE* genome of strains Gauthier and Fribourg-Blanc. *tprD* and *tprK* genes as well as the differences in the number of repeats in *arp* and *TP_0470* genes were excluded from the analysis.

| **Length of nucleotide differences** | **LMNP-1 vs. Gauthier** | **LMNP-1 vs. Fribourg-Blanc** |
| --- | --- | --- |
| 1 nt | 252 | 307 |
| 2 nt | 3 | 5 |
| 3 nt | 1 | 5 |
| 4 nt | 1 | 1 |
| 5 nt | 1 | 0 |
| 6 nt | 0 | 2 |
| 9 nt | 4 | 2 |
| 15 nt | 1 | 1 |
| 33 nt | 1 | 0 |
| 42 nt | 0 | 1 |
| 79 nt | 1 | 0 |
| 302 nt | 1 | 0 |
| 430 nt | 0 | 1 |
| **Total number** | **266** | **325** |
