# Supplementary material for: Nonhuman primates across sub-Saharan Africa are infected with the yaws bacterium Treponema pallidum subsp. pertenue
Source: Emerg Microbes Infect. 2018 Sep 19;7:157. doi: 10.1038/s41426-018-0156-4 (PMC6143531; doi:10.1038/s41426-018-0156-4)
Supplement: Supplementary file 9 — Supplementary Table S8 [file 41426_2018_156_MOESM9_ESM.docx]

**Table S8.** Proteins encoded by the *TPE* strain Fribourg-Blanc genome with 1 and more amino acid changes when compared to the *TPE* strain CDC-2 proteome. *Gene *tprK* (*TPE40M5_0897*) was omitted from the analysis due to its intra-strain variability. ^Δ^Coordinates correspond to the positions leading to amino acid changes. **^○^**Gene was not annotated in the *TPE* strain Fribourg-Blanc genome.

| **Gene***  **Protein [Functional group]** | ***TPE* strain Fribourg-Blanc whole genome coordinates^Δ^** | **Type of change in comparison with *TPE* Gauthier** | **Number of aa changes** | **Result of the frameshift mutation** |
| --- | --- | --- | --- | --- |
| ***TPFB_0005*** *GyrA*, cDP-diacylglycerol--glycerol-3-phosphate 3-phosphatidyltransferase **[**DNA replication, repair, recombination**]** | 6,654 | 1 SNV | 1 |  |
| ***TPFB_0012*** | 12,487-12,488 | 1 bp deletion resulting in frameshift mutation |  | considered as pseudogene, (deletion in position 114 out of 177 bp) |
| ***TPFB_0018*** *GreA*, transcription elongation factor **[**transcription**]** | 20,705 | 1 SNV | 1 |  |
| ***TPFB_0033*** hypothetical protein **[**unknown**]** | 41,224 | 1 SNV | 1 |  |
| ***TPFB_0040*** *Mcp*, putative methyl-accepting chemotaxis protein [cell processes] | 49,369-49,373 | 5 bp insertion resulting in frameshift mutation | 6 | protein shortening on C-terminus from 814 to 810 aa (808 aa similar to *TPE* strain CDC-2) |
| ***TPFB_0092*** *RpoE*, DNA-directed RNA polymerase sigma subunit [transcription] | 101,955 | 1 SNV | 1 |  |
| ***TPFB_0098*** *DnaJ1*, chaperone [cell processes] | 106,884; 107,247 | 2 SNV | 2 |  |
| ***TPFB_0117*** *TprC* [unknown] | within region 134,966-136,550 | 19 SNV | 16 |  |
| ***TPFB_0126a*** hypothetical protein [unknown] | within region 148,979-148,985 | 6 SNV | 2 |  |
| ***TPFB_0126b*** hypothetical protein [unknown] | 148,982-148,985 | 4 SNV | 14 | SNV in START codon, protein shortening on N-terminus from 135 to 121 aa |
| ***TPFB_0131*** *TprD* [unknown] | 153,989 | 1 SNV | 1 |  |
| ***TPFB_0152a*** hypothetical protein [unknown] | 176,235 | 1 SNV | 1 |  |
| ***TPFB_0179*** hypothetical protein [unknown] | 199,635-199,637 | 3-bp insertion | 1 |  |
| ***TPFB_0196*** *RplP*, ribosomal protein L16 [translation] | 210,334 | 1 SNV | 1 |  |
| ***TPFB_0200*** *RplX*, ribosomal protein L24 [translation] | 211,401 | 1 SNV | 1 |  |
| ***TPFB_0236*** *NusG*, transcription antitermination protein [regulation] | 243,855 | 1 SNV | 1 |  |
| ***TPFB_0242*** *RpoC*, DNA-directed RNA polymerase subunit beta prime [transcrition] | 254,357; 254,472 | 2 SNV | 1 |  |
| ***TPFB_0245*** hypothetical protein [unknown] | 258,570 | 1 SNV | 1 |  |
| ***TPFB_0279*** bifunctional cytidylate kinase/ribosomal protein [translation] | 295,340-295,342 | 3-bp insertion | 1 |  |
| ***TPFB_0303*** *MutL*, DNA mismatch repair protein [DNA replication, repair, recombination] | 319,012; 319,401; 321,132 | 3 SNV | 3 |  |
| ***TPFB_0316*** *TprF* [unknown] | within region 332,557-333,448 | 7 SNV | 6 |  |
| ***TPFB_0322*** sugar ABC superfamily ATP binding cassette transporter, membrane protein [transport] | 340,498-340,499; 340,529 | 1-bp deletion and 1-bp insertion in a close proximity | 9 |  |
| ***TPFB_0324*** putative outer membrane protein [unknown] | within region 342,126-345,775 | 5 SNV | 5 |  |
| ***TPFB_0326*** *Tp92*, outer membrane protein [virulence] | 346,413; 348,114; 348,115 | 3 SNV | 2 |  |
| ***TPFB_0344*** *TrcF*, transcription-repair coupling factor [transcription] | 368,400 | 1 SNV | 1 |  |
| ***TPFB_0345a*** hypothetical protein [unknown] | 372,913; 372,920 | 2 SNV | 1 |  |
| ***TPFB_0346*** putative lipoprotein [unknown] | 373,273; 373,352; 373,484 | 3 SNV | 3 |  |
| ***TPFB_0347*** putative membrane protein [unknown] | 373,760; 373,761 | 2-bp insertion leading to frameshift mutation | 40 | protein shortening on N-terminus from 276 to 236 aa |
| ***TPFB_0370*** hypothetical protein [unknown] | 396,159-396,160 | 6-bp deletion | 2 |  |
| ***TPFB_0433*** *arp*, acidic repeat protein [unknown] | 463,017-463,676 | 11x 60-bp insertion | 220 |  |
| ***TPFB_0457*** hypothetical protein [unknown] | 488,933 | 1 SNV | 1 |  |
| ***TPFB_461a*** hypothetical protein [unknown] | 493,022-493,023 | 1-bp deletion resulting in frameshift mutation | 46 | protein elongation on C-terminus from 60 to 81 aa (35 aa similar to *TPE* strain CDC-2) |
| ***TPFB_0462*** putative lipoprotein [unknown] | 493,401-493,403 | 3-bp insertion | 1 |  |
| ***TPFB_0463*** | 494,190 | 1 SNV | 1 |  |
| ***TPFB_0470*** *TPR*domain protein [unknown] | 499,964-499,965 | 15x 24-bp deletion | 120 |  |
| ***TPFB_0484*** hypothetical protein [unknown] | 517,708-517,709 | 1-bp deletion resulting in frameshift mutation | 103 | protein shortening on N-terminus from 671 to 568 aa |
| ***TPFB_0488*** *Mcp*, methyl--accepting chemotaxis protein [cell processes] | within region 523,706-525,356 | 10 SNV | 10 |  |
| ***TPFB_0529*** *NtpA2*, two-sector ATPase, V(1) subunit A [transport] | 575,936 | 1 SNV | 1 |  |
| ***TPFB_0548*** hypothetical protein [unknown] | 594,092-594,093, 594,408; 594,420, 594,421; 595,033 | 42-bp deletion and 4 SNV | 17 |  |
| ***TPFB_0552*** hypothetical protein [unknown] | 600,656 | 1 SNV | 1 |  |
| ***TPFB_0577*** putative membrane protein [unknown] | 630,702; 630,712 | 2 SNV | 2 |  |
| ***TPFB_0584*** hypothetical protein [unknown] | 636,174 | 1 SNV | 1 |  |
| ***TPFB_0596*** *PcnB2*, tRNA polynucleotide adenylyltransferase [translation] | 650,042 | 1 SNV | 1 |  |
| ***TPFB_0620*** *TprI* [unknown] | within region 673,711-674,602 | 8 SNV | 6 |  |
| ***TPFB_0622*** putative membrane protein [unknown] | 679,037 | 1 SNV | 1 |  |
| ***TPFB_0639*** *Mcp*, methyl-accepting chemotaxis protein [cell processes] | 700,639 | 1 SNV | 1 |  |
| ***TPFB_0640*** *Mcp*, methyl-accepting chemotaxis protein [cell processes] | 703,788 | 1 SNV | 1 |  |
| ***TPFB_0652*** *PotA*, spermidine/putrescine ABC superfamily ATP binding cassette transporter, ABC protein [transport] | 718,955 | 1 SNV | 1 |  |
| ***TPFB_0675*** hypothetical protein [unknown] | 743,072 | 1 SNV | 1 |  |
| ***TPFB_0690*** putative lipoprotein [unknown] | 760,088 | 1 SNV | 1 |  |
| ***TPFB_0730*** *PgsA2*, CDP-diacylglycerol--glycerol-3-phosphate 3-phosphatidyltransferase [general metabolism] | 798,522 | 1 SNV | 1 |  |
| ***TPFB_0733*** hypothetical protein [unknown] | 801,328; 801,349 | 2 SNV | 2 |  |
| ***TPFB_0747*** hypothetical protein [unknown] | 814,737 | 1 SNV | 1 |  |
| ***TPFB_0817*** *Eno*, phosphopyruvate hydratase [general metabolism] | 888,576 | 1 SNV | 1 |  |
| ***TPFB_0854*** putative lipoprotein [unknown] | 928,417 | 1 SNV | 1 |  |
| ***TPFB_0856*** putative lipoprotein [unknown] | 936,307; 936,308; 936,310 | 3 SNV | 2 |  |
| ***TPFB_0856a*** hypothetical protein [unknown] | 937,211; 937,317; 937,445; 938,301 | 4 SNV | 4 |  |
| ***TPFB_0858*** putative lipoprotein [unknown] | 937,211; 937,317; 937,445; 938,301 | 4 SNV | 4 |  |
| ***TPFB_0859*** hypothetical protein [unknown] | 938,535; 939,155-939,156 | 1 SNV and 3-bp deletion | 2 |  |
| ***TPFB_0861*** *GlmS*, glutamine-fructose-6-phosphate transaminase [general metabolism] | 946,841; 946,842 | 2 SNV | 1 |  |
| ***TPFB_0865*** putative outer membrane protein [unknown] | within region 946,841-946,848 | 7 SNV | 3 |  |
| ***TPFB_0891*** *InfB*, initiation factor IF2 [translation] | 972,710 | 1 SNV | 1 |  |
| ***TPFB_0896*** | within region 976,990-977,041 | 6 SNV |  | SNV leading to STOP codon, considered as pseudogene |
| ***TPFB_0901*** *NorM*, MATE family multi antimicrobial extrusion protein [transport] | 985,974 | 1 SNV | 1 |  |
| ***TPFB_0949a*** hypothetical protein [unknown] | 1,034,780 | 1 SNV | 1 |  |
| ***TPFB_0952*** putative lipase/esterase [general metabolism] | 1,036,094 | 1 SNV | 1 |  |
| ***TPFB_0957*** *TRAP-T*family tripartite ATP-independent periplasmic transporter [transport] | 1,041,824 | 1 SNV | 1 |  |
| ***TPFB_0960*** *FlgG1*, flagellar basal body rod protein [cell structure] | 1,044,647 | 1 SNV | 1 |  |
| ***TPFB_0966*** putative lipoprotein [unknown] | 1,051,534 | 1 SNV | 1 |  |
| ***TPFB_0967*** hypothetical protein [unknown] | 1,053,139; 1,053,293 | 2 SNV | 1 |  |
| ***TPFB_0968*** hypothetical protein [unknown] | 1,053,676; 1,053,893; 1,054,025; 1,055,233 | 4 SNV | 4 |  |
| ***TPFB_0973*** *PheS*, phenylalanine--tRNA ligase alpha subunit [translation] | 1,059,810 | 1 SNV | 1 |  |
| ***TPFB_0976*** putative membrane protein [unknown] | 1,062,865 | 1 SNV | 1 |  |
| ***TPFB_0993a*** hypothetical protein [unknown] | 1,081,313 | 1 SNV | 1 |  |
| ***TPFB_0998*** *AlsT2*, sodium/alanine symporter family protein [transport] | 1,088,215 | 1 SNV | 1 |  |
| ***TPFB_1007*** *ThyX*, thymidylate synthase [general metabolism] | 1,100,256 | 1 SNV | 1 |  |
