# Supplementary material for: Nonhuman primates across sub-Saharan Africa are infected with the yaws bacterium Treponema pallidum subsp. pertenue
Source: Emerg Microbes Infect. 2018 Sep 19;7:157. doi: 10.1038/s41426-018-0156-4 (PMC6143531; doi:10.1038/s41426-018-0156-4)
Supplement: Supplementary file 10 — Supplementary Table S9 [file 41426_2018_156_MOESM10_ESM.docx]

**Table S9.** Proteins encoded by the LMNP-1 baboon strain with one and more amino acid changes when compared to the *TPE* strain Gauthier proteome. *Gene *tprK* (*TPE40M5_0897*) was omitted from the analysis due to its intra-strain variability. ^Δ^Coordinates correspond to the positions leading to amino acid changes.

| **Gene***  **Protein [Functional group]** | **40M5160407 whole genome coordinates^Δ^** | **Type of change in comparison with *TPE* strain Gauthier** | **Number of aa changes** | **Result of the frameshift mutation** |
| --- | --- | --- | --- | --- |
| ***TPE40M5_0001*** *DnaA*, DNA-directed DNA replication initiator protein [DNA replication, repair, recombination] | 263 | 1 SNV | 1 |  |
| ***TPE40M5_0006*** putative lipoprotein [unknown] | 7,547-7,548; 8,213 | 1-bp deletion resulting in frameshift mutation, 1 SNV | 237 | protein shortening on C-terminus from 415 to 195 aa (178 aa similar to *TPE* strain Gauthier) |
| ***TPE40M5_0012*** hypothetical protein [unknown] | 12,486-12,487 | 1-bp deletion resulting in frameshift mutation | 20 | protein shortening on C-terminus from 58 to 42 aa (38 aa similar to *TPE* strain Gauthier) |
| ***TPE40M5_0023*** *NSS* family putative amino acid:sodium (Na+) symporter [transport] | 27,869 | 1 SNV | 1 |  |
| ***TPE40M5_0040*** *Mcp*, putative methyl-accepting chemotaxis protein [cell processes] | 49,368-49,371 | 5-bp insertion resulting in frameshift mutation | 6 | protein shortening on C-terminus from 771 to 767 aa (765 aa similar to *TPE* strain Gauthier) |
| ***TPE40M5_0042*** hypothetical protein [unknown] | 49,826 | 1 SNV | 1 |  |
| ***TPE40M5_0067*** *TPR*domain protein [unknown] | 72,691-72,694 | 4-bp insertion resulting in frameshift mutation | 11 | protein shortening on N-terminus from 390 to 376 aa |
| **-** | 72,732-72,733 | 9-bp deletion | 3 |  |
| **-** | 72,862; 72,952 | 2 SNV | 2 |  |
| ***TPE40M5_0083*** hypothetical protein [unknown] | 93,789 | 1 SNV | 1 |  |
| ***TPE40M5_0098*** *DnaJ*chaperone [cell processes] | 107,102 | 1 SNV | 1 |  |
| ***TPE40M5_0117*** *TprC* [unknown] | 136,694-136,695 | 1-bp deletion |  | deletion in START codon, instead of GTG gene starts with ATG |
| **-** | within region 134,911-136,545 | 26 SNV | 15 |  |
| ***TPE40M5_0119*** methionine ABC superfamily ATP binding cassette transporter [transport] | 138,272 | 1 SNV | 1 |  |
| ***TPE40M5_0127a*** hypothetical protein [unknown] | 150,210-150,211 | 1-bp insertion resulting in frameshift mutation | 106 | protein elongation on C-terminus from 126 to 222 aa (116 aa similar to *TPE* strain Gauthier) |
| ***TPE40M5_0127b*** putative lipoprotein [unknown] | 150,210-150,211; 150463 | 1-bp insertion resulting in frameshift mutation and 1 SNV |  | considered as pseudogene (insertion in position 74-75 out of 414 bp) |
| ***TPE40M5_0131*** *TprD* [unknown] | 154,140-154,141 | 1-bp deletion |  | deletion in START codon, instead of GTG gene starts with ATG |
| ***-*** | within region 152,357-153,408 | 25 SNV | 14 |  |
| ***TPE40M5_0134*** putative outer membrane protein [unknown] | 156,213-156,215; 156,253 | 3-bp insertion and 1 SNV | 2 |  |
| ***TPE40M5_0136*** putative outer membrane protein [virulence] | 158,538-158,570 | 33-bp insertion | 11 |  |
| **-** | within region 158,132-158,511 | 7 SNV | 3 |  |
| ***TPE40M5_0143*** ABC superfamily ATP binding cassette transporter, membrane protein [transport] | 164,599 | 1 SNV | 1 |  |
| ***TPE40M5_0219*** probable sigma factor regulatory protein [regulation] | 224,855; 226,013 | 2 SNV | 2 |  |
| ***TPE40M5_0230*** *PriA*, DNA replication factor Y [DNA replication, repair, recombination] | 239,958 | 1 SNV | 1 |  |
| ***TPE40M5_0249*** *FlaA*, flagellar filament outer layer protein [cell structure] | 262,681 | 1 SNV | 1 |  |
| ***TPE40M5_0259*** *LysM*domain protein [unknown] | 271,476-271,484 | 9-bp insertion | 3 |  |
| ***TPE40M5_0279*** bifunctional cytidylate kinase/ribosomal protein [translation] | 296,015; 296193 | 2 SNV | 1 |  |
| ***TPE40M5_0286*** hypothetical protein [unknown] | 301,572 | 1 SNV | 1 |  |
| ***TPE40M5_0312a*** hypothetical protein [unknown] | 329,051-329,052 | 1-bp deletion resulting in frameshift mutation |  | considered as pseudogene (deletion in position 103-104 out of 162 bp) |
| ***TPE40M5_0313*** *TprE* [unknown] | 330,487; 331,148; 331,175; 331,206 | 4 SNV | 2 |  |
| ***TPE40M5_0316*** *TprF* [unknown] | 334,039-334,040 | 1-bp deletion | 1 | deletion in START codon, instead of GTG gene starts with ATG |
| ***-*** | within region 332,289-334,033 | 24 SNV | 18 |  |
| ***TPE40M5_0317*** *TprG* [unknown] | 334,965 | 1 SNV | 1 |  |
| ***TPE40M5_0319*** *TmpC*, sugar ABC superfamily ATP binding cassette transporter, membrane protein [transport] | 336,858; 336,869; 336,963 | 3 SNV | 3 |  |
| **-** | 337,590 | 1 SNV |  | read through STOP codon, fusion to *TPE40M5_0320* |
| ***TPE40M5_0321*** sugar ABC superfamily ATP binding cassette transporter [transport] | 337,867 | 1 SNV | 1 |  |
| ***TPE40M5_0326*** *Tp92*, outer membrane protein [virulence] | within region 347,585-348,193 | 7 SNV | 7 |  |
| ***TPE40M5_0334*** *HTH* domain protein [unknown] | 359,096 | 1 SNV | 1 |  |
| ***TPE40M5_0346*** putative lipoprotein [unknown] | 373,022; 373,479; 373,488 | 3 SNV | 2 |  |
| ***TPE40M5_0347*** putative membrane protein [unknown] | 373,756-373,757 | 2-bp insertion resulting in frameshift mutation | 40 | protein shortening on N-terminus from 276 to 236 aa |
| ***TPE40M5_0398*** *FliE* flagellar hook-basal body protein [cell structure] | 424,529 | 1 SNV | 1 |  |
| **TPE40M5_0401** *FliH*, IIISP family Type III (virulence-related) secretory pathway protein [virulence] | 428,180 | 1 SNV | 1 |  |
| ***TPE40M5_0433*** *arp*, acidic repeat protein [unknown] | 462,316 | 1 SNP | 1 |  |
| **-** | 463,318-463,319 | 1x 60-bp deletion | 20 |  |
| ***TPE40M5_0444*** *LysM* domain protein [unknown] | 472,228 | 1-bp insertion resulting in frameshift mutation | 42 | protein shortening on N-terminus from 342 to 300 aa |
| ***TPE40M5_0462*** putative lipoprotein [unknown] | 493,896-493,897 | 9-bp deletion | 3 |  |
|  | 493,749 | 1 SNP | 1 |  |
| ***TPE40M5_0483*** hypothetical protein [unknown] | 514,807 | 1 SNV | 1 |  |
| ***TPE40M5_0488*** *Mcp*, methyl-accepting chemotaxis protein [cell processes] | within region 523,457-524,502 | 11 SNV | 11 |  |
| ***TPE40M5_0496*** *TPR* domain protein [unknown] | 533,718 | 1 SNV | 1 |  |
| ***TPE40M5_0505*** hexokinase [general metabolism] | 541,183 | 1 SNV | 1 |  |
| ***TPE40M5_0512*** *IspDF*, bifunctional 2-C-methyl-D-erythritol 4-phosphate cytidylyltransferase/2-C-methyl-D-erythritol 2,4-cyclodiphosphate synthase [general metabolism] | 550,259; 550,820 | 2 SNV | 2 |  |
| ***TPE40M5_0514*** *UvrA*, excision endonuclease subunit [DNA replication, repair, recombination] | 552,914 | 1 SNV | 1 |  |
| ***TPE40M5_0526*** *HrpA*, ATP-dependent helicase [DNA replication, repair, recombination] | 571,132 | 1 SNV | 1 |  |
| ***TPE40M5_0528*** *NtpB2*, two-sector ATPase, V(1) subunit B [transport] | 573,806 | 1 SNV | 1 |  |
| ***TPE40M5_0534*** hypothetical protein [unknown] | 579,146; 579,341 | 2 SNV | 2 |  |
| ***TPE40M5_0546*** *S1B* subfamily peptidase [general metabolism] | 591,706 | 1 SNV | 1 |  |
| ***TPE40M5_0548*** outer membrane protein [unknown] | within region 593,836-594,613 | 7 SNV | 7 |  |
| ***TPE40M5_0549*** *ClpA*, S14 family endopeptidase [cell processes] | 595,254 | 1 SNV | 1 |  |
| ***TPE40M5_0569*** Xaa-Pro aminopeptidase [general metabolism] | 620,521 | 1 SNV | 1 |  |
| ***TPE40M5_0584*** hypothetical protein [unknown] | 636,180 | 1 SNV | 1 |  |
| ***TPE40M5_0610*** *TprH* [unknown] | 664,542-664,543 | 1-bp deletion resulting in frameshift mutation | 280 | protein shortening from 693 to 475 aa (413 aa similar to *TPE* strain Gauthier) |
| ***TPE40M5_0611*** ABC superfamily ATP binding cassette transporter, ABC protein [transport] | 666,233 | 1 SNV | 1 |  |
| ***TPE40M5_0620*** *TprI* [unknown] | 674,940-674,941 | 1-bp deletion | 1 | deletion in START codon, instead of GTG gene starts with ATG |
| **-** | within region 673,190-674,934 | 24 SNV | 18 |  |
| ***TPE40M5_0621*** *TprJ* [unknown] | 675,865 | 1 SNV | 1 |  |
| ***TPE40M5_0622*** putative membrane protein [unknown] | 677,330 | 1 SNV | 2 | SNV leading to STOP codon, protein truncation on C-terminus from 595 to 593 aa |
| ***TPE40M5_0629*** hypothetical protein [unknown] | 689,822 | 1 SNV | 1 |  |
| ***TPE40M5_0639*** methyl-accepting chemotaxis protein [cell processes] | 700,841; 701,394 | 2 SNV | 2 |  |
| ***TPE40M5_0640*** *Mcp*, methyl-accepting chemotaxis protein [cell processes] | 702,455; 702,637; 702,679 | 3 SNV | 3 |  |
| ***TPE40M5_0652*** *PotA*, spermidine/putrescine ABC superfamily ATP binding cassette transporter [transport] | 717,776 | 1 SNV | 1 |  |
| ***TPE40M5_0654*** *PotC*, spermidine/putrescine ABC superfamily ATP binding cassette transporter [transport] | 719,981; 720,341; 720;540 | 3 SNV | 3 |  |
| ***TPE40M5_0678*** hypothetical protein [unknown] | 744,875 | 1 SNV | 1 |  |
| ***TPE40M5_0684*** *MglB*, galactose ABC superfamily ATP binding cassette transporter, binding protein [transport] | 750,550 | 1 SNV | 1 |  |
| ***TPE40M5_0693*** putative lipoprotein [unknown] | 762,579 | 1 SNV | 1 |  |
| ***TPE40M5_0696*** putative nicotinamidase [general metabolism] | 767,109 | 1-bp insertion resulting in frameshift mutation | 20 | protein shortening on N-terminus from 278 to 258 aa |
| ***TPE40M5_0698*** hypothetical membrane protein [unknown] | 768,282 | 1 SNV | 1 |  |
| ***TPE40M5_0722*** *FliL2*, flagellar basal body-associated protein [cell structure] | 792,057 | 1 SNV | 1 |  |
| ***TPE40M5_0733*** hypothetical protein [unknown] | 800,658; 800,673; 800,717 | 3 SNV | 3 |  |
| ***TPE40M5_0741*** *NadD*, putative nicotinate-nucleotide adenylyltransferase [general metabolism] | 808,690 | 1 SNV | 1 |  |
| ***TPE40M5_0746*** *PpdK*, pyruvate, phosphate dikinase [general metabolism] | 812,279; 813,232 | 2 SNV | 2 |  |
| ***TPE40M5_0748*** *CfpA*, cytoplasmic filament protein A [cell processes] | 816,355 | 1 SNV | 1 |  |
| ***TPE40M5_0758*** *RpsU*, ribosomal protein S21 [translation] | 824,067 | 1 SNV | 1 |  |
| ***TPE40M5_0761*** hypothetical protein [unknown] | 827,081-827,082 | 2-bp deletion resulting in frameshift mutation |  | fusion to *TPE40M5_0762* |
| ***TPE40M5_0764*** HD-GYP domain protein [regulation] | 830,136 | 1 SNV | 1 |  |
| ***TPE40M5_0796*** putative *ApbE* family protein [general metabolism] | 864,688; 865,092 | 2 SNV | 1 |  |
| ***TPE40M5_0804*** sugar ABC superfamily ATP binding cassette transporter [transport] | 874,803 | 1 SNV | 1 |  |
| ***TPE40M5_0842*** *Map*, methionyl aminopeptidase [translation] | 917,314 | 1 SNV | 1 |  |
| ***TPE40M5_0856a*** hypothetical protein [unknown] | within region 936,623-936,821 | 6 SNV | 6 |  |
| **TPE40M5_0858** putative lipoprotein [unknown] | 937,531-937,609 | 79-bp insertion resulting in frameshift mutation | 49 | protein elongation on C-terminus from 385 to 409 aa (360 aa similar to *TPE* strain Gauthier) |
| **-** | within region 936,623-937,432 | 8 SNV | 6 |  |
| ***TPE40M5_0859*** hypothetical protein [unknown] | 938,460-938,461 | 9-bp deletion | 3 |  |
| **-** | 938,313 | 1 SNP | 0 |  |
| ***TPE40M5_0865*** putative outer membrane protein [unknown] | 945,781; 946,149 | 2 SNP | 2 |  |
| ***TPE40M5_0896*** hypothetical protein [unknown] | 976,339; 976,341; 976,342; 976,344 | 4 SNP |  | SNV leading to STOP codon, considered as pseudogene |
| ***TPE40M5_0898*** *RecB*, exodeoxyribonuclease V beta subunit [DNA replication, repair, recombination] | 977,954 | 1-bp insertion resulting in frameshift mutation | 7 | protein truncation from 1,239 to 1,238 aa (1,232 aa similar to *TPE* strain Gauthier) |
| ***TPE40M5_0919*** thioredoxin group 1 family protein [general metabolism] | 998,374; 998,555 | 2 SNV | 2 |  |
| ***TPE40M5_0931*** hypothetical protein [unknown] | 1,015,467 | 1 SNV | 1 |  |
| ***TPE40M5_0939*** pyruvate synthase [general metabolism] | 1,025,096 | 1 SNV | 1 |  |
| ***TPE40M5_0949*** *Oxa1*, family cytochrome oxidase biogenesis protein [transport] | 1,032,124 | 1 SNV | 1 |  |
| ***TPE40M5_0952*** putative lipase/esterase [general metabolism] | 1,035,289; 1,035,290 | 2 SNV | 1 |  |
| ***TPE40M5_0966*** thioredoxin group 1 family protein [general metabolism] | 1,049,816 | 1 SNV | 1 |  |
| ***TPE40M5_0967*** hypothetical protein [unknown] | 1,052,396; 1,052,398 | 2 SNV | 1 |  |
| ***TPE40M5_0968*** hypothetical protein [unknown] | 1,053,293 | 1 SNV | 1 |  |
| ***TPE40M5_0969*** putative outer membrane protein [unknown] | 1,055,256 | 1 SNV | 1 |  |
| ***TPE40M5_0976*** hypothetical protein [unknown] | 1,062,454-1,062,468 | 15-bp insertion | 5 |  |
| ***TPE40M5_1012*** *RpoD*, DNA-directed RNA polymerase sigma subunit [transcription] | 1,105,470 | 1 SNV | 1 |  |
| ***TPE40M5_1035*** valine--tRNA ligase [translation] | 1,130,609; 1,132,018 | 2 SNV | 1 |  |
