# Supplementary material for: Nonhuman primates across sub-Saharan Africa are infected with the yaws bacterium Treponema pallidum subsp. pertenue
Source: Emerg Microbes Infect. 2018 Sep 19;7:157. doi: 10.1038/s41426-018-0156-4 (PMC6143531; doi:10.1038/s41426-018-0156-4)
Supplement: Supplementary file 11 — Supplementary Materials [file 41426_2018_156_MOESM11_ESM.docx]

**Supplementary materials**

**Laboratory contributions.** The study brought together NHP samples from four field sites and involved analysis conducted in multiple different laboratories. As a consequence of this, several analysis steps (e.g., DNA extraction, PCR testing, DNA capture, sequencing and genome assembly) were performed with different methods. Briefly, samples from West Africa were collected by the University of Cumbria (Carlisle, UK), the Research Unit of Emerging Infectious and Tropical Diseases (Marseilles, France and Dakar, Senegal: URMITE) and the group Epidemiology of Highly Pathogenic Microorganisms (Robert Koch Institute, Berlin, Germany: RKI) and processed by URMITE and RKI. Samples from East Africa were collected and processed by the Work Group Neglected Tropical Diseases (German Primate Center, Göttingen, Germany: DPZ). While DNA samples from West Africa were analyzed by in-solution capture at RKI and by microarray capture at the Department of Archaeological Sciences (Eberhard-Karls-University Tuebingen, Germany: EKU), samples from East Africa were only microarray captured at EKU. In addition, East African samples were used to run long-range PCR followed by next-generation sequencing at the Department of Biology (Masaryk University, Brno, Czech Republic: MU). Obtained sequence data from all samples were collected and shared between collaborators and jointly analyzed at EKU.

**Sampling procedures in NHPs.** Seven clinically affected and four unaffected individuals from the TaïNP and BFP groups were anesthetized using a combination of ketamine (10mg/kg)/xylazine (1mg/kg) (at TCP) or ketamine (5mg/kg)/medetomidine (50µg/kg) (at BFP) administered intramuscularly via blowpipe (Telinject GmbH; TCP group) or by hand-injection after trapping (BFP group). An intramuscular injection of atipamezole (1mg/10 mg xylazine or 5mg/1mg medetomidine) was administered after sampling for reversal of anesthesia. Skin biopsies and blood samples were collected from all individuals. Skin biopsies were taken from the edge of a lesion in clinically affected individuals and from normal unaffected tissue in both clinically affected and healthy individuals. Skin biopsies were preserved in a 10 % formalin solution, in a formaldehyde/glutaraldehyde mixture and/or frozen (here and subsequently this refers to freezing in liquid nitrogen in the field and at -80°C in the laboratory). Where refrigeration was not immediately available (BFP group), samples were preserved in RNAlater (Life technologies, NY) and kept at room temperature for three weeks, then transferred to -80°C. Blood was collected in EDTA tubes from the femoral vein. Whole blood was preserved frozen (TCP group) or in RNALater (BFP group). For the TCP group, swabs from lesions preserved in STGG transport medium were also collected and refrigerated until analysis. The two baboons at LMNP were immobilized using an intramuscular injection of ketamine (10mg/kg)/xylazine (0.2mg/kg). Skin samples were taken from the margin of ulcerated genital tissue, using a sterile 6 mm biopsy punch (Heiland VET Vertriebsgesellschaft mbH & Co. KG, Hamburg, Germany). Skin samples were immediately transferred into RNAlater and were frozen 12 hours later at -20°C until export to Germany. Swabs of penile lesions from African green monkeys in Senegal were transported without culture medium in liquid nitrogen.

**Ethics statement.** All procedures performed on sooty mangabeys in TaïNP were approved by the Ministry of Environment and Forests as well as the Ministry of Research, the Office Ivoirien des Parcs et Réserves, and the director of TaïNP. Baboon samples from LMNP in Tanzania were taken in accordance with the Tanzania Wildlife Research Institute’s Guidelines for Conducting Wildlife Research (2001; 2012) and with permission of Tanzania National Parks (TNP/HQ/E.20/08B) as well as the Commission for Science and Technology in Tanzania (2007-56-NA-2006-176). The Joint Management Research Committee (JMRC) of the TAWIRI Board and TANAPA approved sample collection. The Animal Welfare and Ethics Committee of the German Primate Center approved the use of samples for this study. Procedures on green monkeys in BFP were conducted with ethical approval from the University of Cumbria and permission from the Forestry Department and Department of Parks and Wildlife Management in The Gambia. African green monkeys were captured in Niokolo Koba National Park (NKNP), Senegal, under license No. 1302/DPN/MEDD (10.16.2015) granted by the Republic of Senegal. Good veterinary practice and animal welfare were considered in all procedures carried out at all field sites. Anesthetized animals were monitored for vital functions and remained under close supervision from the time of induction until full recovery, and until the animals were able to reunite with their social group.

**DNA extraction.** At the RKI, DNA was extracted from tissue, blood and swabs using DNA/RNA purification kits (MACHEREY-NAGEL GmbH & Co. KG, Düren, Germany), QIAamp DNA Blood Mini Kits (QIAGEN GmbH, Hilden, Germany) and QIAamp Viral RNA Mini Kits (QIAGEN GmbH, Hilden, Germany) respectively. DNA extraction from LMNP baboon samples was performed twice (18). Tissue was cut into small pieces and ground with Precillys-Keramik beads (peQlab Biotechnologie GmbH, Erlangen, Germany), followed by 4-6 hours of digestion with proteinase Kat 56°C. The first extraction (in 2007) was performed using the NucleoSpin Tissue extraction kit (MACHEREY-NAGEL GmbH & Co. KG, Düren, Germany), the second extraction (in 2014) using the First-DNA-All-Tissue kit (Gen-ial GmbH, Troisdorf, Germany). In both cases, DNA was eluted in molecular grade water. Aliquots were kept frozen at -80°C.

**PCR testing.** Tissue and blood samples (TCP, BFP and NKNP groups) were screened for *TP* infections using PCR and subsequent Sanger’s sequencing (**Table S1**). Extracts were tested using a standard PCR amplifying a 67-bp DNA fragment (includes primers) of the DNA polymerase I gene (*polA*) as previously described (18, 42). Amplifications were performed in 25 µl reactions containing 0.2 µM of each primer, 200 µM dNTPs, 4 mM MgCl_2,_ 2,5 µl 10X PCR buffer and 0.25 µl Platinum® Taq polymerase (Invitrogen, Carlsbad, USA). Assays were run under the following conditions: 5 min at 95°C, 40 cycles [15 s at 95°C, 45 s at 60°C, 60 s at 72°C], 7 min at 72°C.

**qPCR testing.** *TP* copy numbers in the LMNP baboon samples were measured using a TaqMan PCR targeting the same 67-bp fragment of the DNA polymerase I gene (*polA*) of *T. pallidum* (42) and run on a 7500 Real Time PCR System (Applied Biosystems Inc., Foster City, CA, USA). A dilution series of a plasmid containing the target amplicon was used as a standard. Cloning was performed using the TOPO TA cloning kit (Invitrogen, Karlsruhe, Germany) and plasmid preparation with the NucleoSpin Plasmid kit (MACHEREY-NAGEL GmbH & Co. KG, Düren, Germany). Samples were tested in duplicate in 2007 and triplicate in 2014. For the individual 4F5230307, copy numbers were 4,696±1,824/100 ng genomic DNA (gDNA; mean±SD; DNA extraction in 2007) and 2±1/100 ng gDNA in DNA extracted in 2014. Likewise, DNA extracted in 2007 from animal 40M5160407 revealed a copy number of 7,250/100 ng gDNA and 102±2/100 ng gDNA copies (mean±SD) for the DNA extracted in 2014. The low copy numbers detected in DNA that was extracted in 2014 could be an effect of different extraction methods or of the unequal distribution of spirochetes in skin samples. The first DNA extraction was performed immediately after samples were imported to Germany in 2007 and DNA degradation might also be responsible for differences observed between these time periods.

**DNA amplification, capture, and sequencing.** Three different approaches were used to amplify and sequence the whole genomes of the simian TP strains. The West African TP strains (TaïNP-1 and 2, BFP-1 and -2, NKNP-1 and -2) were whole genome sequenced using in-solution and microarray based hybridization capture, for the East African TP strains LMNP-1 and 2 we only used microarray based hybridization capture prior to whole genome sequencing. The LMNP-1 strain was also sequenced using long-range PCR, allowing us to resolve multiple repeat and paralogous regions where hybridization capture approaches proved less effective. Sequencing was performed on Illumina platforms.

***Long-range PCR products.*** The purified genomic DNA of strain LMNP-1 was amplified using the multiple displacement amplification approach (REPLI-g kit, QIAGEN, Valencia, CA, USA) according to the manufacturer’s instructions. Resulting DNA was 100x diluted and used for the pooled segment genome sequencing (PSGS) as described previously (17, 25). Briefly, DNA was amplified with 278 pairs of specific primers to obtain overlapping PCR products (**Table S4)** covering the entire genome of the LMNP-1 isolate. PCR products were amplified with PrimeSTAR GXL DNA Polymerase (Takara Bio Inc., Otsu, Japan) using touchdown PCR. The cycling conditions were: denaturation at 94°C for 1 min; 8 cycles: 98°C for 10 s, 68-60°C for 15 s (annealing temperature gradually reduced by 1°C/every cycle), and 68°C for 6 min; 35 cycles: 98°C for 10 s, 61°C for 15 s, and 68°C for 6 min (43 cycles in total); followed by the final extension at 68°C for 7 min. PCR products were purified using a QIAquick PCR Purification Kit (QIAGEN, Valencia, CA, USA) according to manufacturer's instructions. To facilitate sequencing of RNA operons, *tpr* genes, and other paralogous regions, PCR products were split equimolarly into four distinct pools. Prior to next-generation sequencing on a MiSeq platform (Illumina), these pools were labeled with multiplex identifier (MID) adapters and sequenced as four different samples.

***Microarray capture.*** DNA extracts were sheared with a Covaris S220 Focused-ultrasonicator® to produce fragments between 300 and 500 bp using the following conditions: 5 intensity, 200 cycles per burst, 45 seconds. Samples were then each concentrated to 30 μl with Amicon® Ultra Centrifugal Filters (Merck KGaA, Darmstadt, Germany) using the manufacturer’s instructions and then converted into double-stranded Illumina libraries as described by Meyer and Kircher, 2010 (43). Sample specific barcodes were added to both library adapters to obtain double indexed libraries (44). Library blanks were treated accordingly. The efficiency of reactions was tested using a quantification assay with the primer set IS5 and IS6 (43), the DyNAmo Flash SYBR Green qPCR Kit (Biozym Scientific GmbH, Hessisch Oldendorf, Germany), and the Lightcycler® 96 (Roche Life Science). For all indexed libraries, a second amplification was performed in 100 µl reactions containing 5 µl library template, 4 units AccuPrime Taq DNA Polymerase High Fidelity (Invitrogen), 1 unit 10X AccuPrime buffer (containing dNTPs) and 0.3 µM IS5 and IS6 primers (43), with the following thermal profile: 2-min initial denaturation at 94°C, followed by five to 18 cycles consisting of 30-sec denaturation at 94°C, a 30-sec annealing at 60°C and a 2-min elongation at 68°C and a 5-min final elongation at 68°C. Purification of the amplified products was performed using MinElute spin columns (QIAGEN GmbH, Hilden, Germany) following the manufacturer’s protocol. For hybridization capture, amplified indexed libraries were quantified with an Agilent Bioanalyzer DNA 1000 Chip and pooled in equimolar amounts. Treponemal DNA was enriched from equimolarly pooled libraries via two rounds of hybridization capture using 1 million Agilent SureSelect arrays with designed probes (60 bp length and 4 bp tiling density) that span the *T. pallidum* genome (1) following the protocol described by Hodges et al., 2009 (45). The first round of hybridization was followed by elution of capture products in 490 µl H_2_O, quantification via qPCR (see previous section), and amplifications in 100 µl reactions using 24 µl template (reagents and thermo profile as described previously). Amplified products were purified using MinElute columns (Qiagen) and concentrations were determined using an Agilent 2100 Bioanalyzer DNA 1000 chip. Amplified products were then used for the second round of capture, after which captured products were again eluted in 490 µl H_2_O and processed as described above with 48 µl template for amplification. Afterwards, captured products were quantified and diluted to 10 nM for high-throughput sequencing. Paired-end dual index sequencing was conducted on an Illumina Hiseq 2500 platform using 2x100+7+7 cycles and manufacturer’s protocols for multiplex sequencing (TruSeq PE Cluster Kit v3-cBot-HS).

***In-solution capture.*** Selected PCR positive extracts were fragmented using a Covaris S220 Focused-ultrasonicator® in a total volume of 130 µL (filled with low EDTA TE buffer), using settings aiming to generate approximately 400-bp fragments (intensity=4, duty cycle=10%, cycles per burst=200, treatment time=55 seconds, temperature=7°C). Fragmented extracts were then concentrated using a MinElute PCR purification kit and each fragmented extract was eluted into 2x10 µL low EDTA TE buffer. DNA concentration was measured using a Qubit dsDNA High Sensitivity kit. 1μg DNA or all available remaining DNA extract were used for subsequent library preparation using the Accel-NGS 2S DNA library kit following the standard protocol and a sample specific unique index. Quantification was conducted using a KAPA HiFi library quantification kit and libraries were then amplified using a KAPA Hot Start Library Amplification Kit and Illumina adapter specific primers (5'-3': AATGATACGGCGACCACCGA and 5'-3': CAAGCAGAAGACGGCATACGA, 45 s at 98°C, variable number of cycles [15 s at 98°C, 30 s at 65°C, 45 s at 72°C], 1 min at 72°. Following amplification libraries were requantified to ensure the desired amount of starting material for capture (total number of cycles before 1^st^ capture: HATO=3 cycles, M3 and IGU=4 cycles, A10 and A12=5 cycles, A9 and M2=6 cycles). HATO, IGU, M2 and M3 were pooled to equally contribute to a total of 240 ng DNA of starting material for input into the in-solution hybridization capture, following concentration with a MinElute PCR Purification Kit (pool A). A9, A10 and A12 were pooled to contribute equally to 500 ng DNA after concentration with a MinElute PCR Purification Kit (pool B). These two pools underwent separate hybridization capture and sequencing at RKI. We designed RNA baits to span the *T. p. pertenue* Fribourg-Blanc genome (accession number NC_021179) with 2-fold tiling and 120mer baits, and used these for hybridization capture enrichment of *TP* DNA as described previously (12). Briefly, we followed the Mybaits Sequence Enrichment for Targeted Sequencing protocol (Version 2.3.1) using the recommended hybridization time of 16 hours. Following an initial round of capture, pools of surviving libraries were reamplified using a KAPA Hot Start Library Amplification Kit (pool A=18 cycles, pool B=15 cycles) to generate 100-500 ng starting material for a second round of capture. Pools were again quantified using the KAPA HiFi Library Quantification Kit to determine concentration of libraries. DNA was concentrated using a MinElute PCR Purification Kit prior to commencing with a second round of hybridization capture applying the same conditions as for the first round of capture described above. Surviving DNA was reamplified using the KAPA Hot Start Library Amplification Kit (pool A=10 cycles, pool B=13 cycles), purified using the MinElute PCR Purification Kit, and quantified using the KAPA HiFi Library Quantification Kit. The two pools were diluted to 4nM as input for two separate sequencing runs on an Illumina MiSeq (v3 2x300 Chemistry).

**In silico analysis.** We applied EAGER^1^, a comprehensive pipeline for read pre-processing, mapping, variant identification, and genome reconstruction to all samples from which genome-wide information was obtained using hybridization capture. Paired-end raw reads were first pre-processed, including adapter clipping, merging of corresponding paired-end reads in the overlapping regions, and finally quality trimming. To remove sequencing adapters from the paired-end reads, an overlap alignment of the respective adapter with the 3’ end of each forward and reverse read was produced. Regions at the 3’ end of each read that were contained in the alignment were clipped. Reads that were shorter than 30 nucleotides after adapter clipping were removed. This resulted in three kinds of remaining reads: forward reads that did not have a corresponding reverse read, reverse reads that did not have a corresponding forward read, and matching forward and reverse reads that could be used in the merging process. Merging was performed for all paired-end reads with a minimum overlap of 10 nucleotides and at most 5% mismatches in the overlap region with the Clip&Merge tool implemented in EAGER. On average, it was possible for approximately 60% of paired-end reads to be merged in each sample. All reads that could not be merged were first trimmed at the 3’ end such that all bases had a phred quality score of at least 20 and were then mapped individually. The resulting reads (merged and unmerged) were treated as single-end reads and mapped using the BWA-MEM algorithm^2^ with default parameters and Fribourg-Blanc as a reference sequence. After mapping the Genome Analysis Toolkit (GATK)^3^ was used to generate a mapping assembly for each strain that had at least 80% coverage of the Fribourg-Blanc genome with a minimum of 3 reads. For this procedure, the UnifiedGenotyper module of GATK was applied to call reference bases and variants from the mapping. A reference base was called if the position was covered by at least three reads and the genotype quality of the call was at least 30. A variant position (single nucleotide polymorphism; SNP) was called if the following criteria were met: i) the position was covered by at least three reads; ii) the genotype quality of the call was at least 30, and iii) the minimum SNP allele frequency was 90%. If the requirements for a variant call were not fulfilled, the reference base was called only if at least 3 reads confirmed it and the quality threshold was reached. If neither of the requirements for a reference base call nor the requirements for a variant call were met, the character ’N’ was inserted at the respective position. Draft genome sequences were generated using the tool VCF2Genome of the EAGER pipeline.

***Read preprocessing of sequenced genome samples.*** We then built a reference data set for phylogenomic analysis. We used short read archives for samples recently analyzed by an extensive study of human TP diversity^4^. Raw reads were processed as described above and we only included genome sequences which achieved 80% coverage of the Fribourg-Blanc genome with a minimum of 3 reads, and corresponded to Nichols-like TPA (i.e. within the Nichols clade) or TPE strains (S3 Table). We also used the completely assembled genomic sequences already available in GenBank (S3 Table), from which we first generated artificial reads using the tool Genome2Reads (also part of the EAGER pipeline). Genome2Reads uses a tiling approach with an offset of 1 to artificially generate reads of 100nt reads, resulting in an average coverage of 100x. We applied the same mapping, SNP calling and genome reconstruction procedure as for short read-based analysis. The genomes reconstructed in our study and the reference genomes were therefore determined in a consistent manner, ensuring comparable results for phylogeny reconstruction.

***Phylogenomic analysis.*** We performed phylogenetic analyses in maximum parsimony (MP), maximum likelihood (ML) and Bayesian frameworks. The analysis involved 29 nucleotide sequences, including 8 NHP strains from this study, 1 NHP strain already available and 20 human strains (**S3 Table**). After alignment, all positions with less than 85% site coverage were eliminated; in other words, fewer than 15% alignment gaps, missing data, and ambiguous bases were allowed at any position; all positions from putative recombinant genes were also removed^4^. MEGA6 was used to generate a MP tree (**S1A Fig**)^5^. Bootstrap values were inferred from 100 pseudo-replicates. For probabilistic methods, the best model of nucleotide substitution was first determined using ModelFinder^6^ as implemented in IQ-TREE^7^. The ML tree was identified using IQ-TREE and branch robustness was estimated using ultrafast bootstrap approximation (**Fig 1**). Bayesian analyses were performed using MrBayes^8^. Multiple Bayesian Monte Carlo Markov chains were run. Convergence and appropriate sampling of the posterior were checked using Tracer (<http://tree.bio.ed.ac.uk/software/tracer/>). The final posterior sample of trees was summarized on the maximum clade credibility tree (**S1B Fig**). Branch robustness was assessed with posterior probabilities.

***Genomic structure analysis.*** The Illumina sequencing reads obtained from the 4 distinct pools of long-range PCR products derived from LMNP-1 (sequenced as 4 different samples; **S2** and **S5 Tables**) were separately assembled *de novo* using SeqMan NGen v4.1.0 software (DNASTAR, Madison, WI, USA). A total of 99, 81, 62, and 138 contigs (obtained for pools 1, 2, 3, and 4, respectively) were aligned to the corresponding sequences of the reference Fribourg-Blanc genome ^9^ using Lasergene software (DNASTAR, Madison, WI, USA). In addition, Illumina sequencing reads were also mapped to the Fribourg-Blanc genome and processed as described above. All gaps in the genome sequence and discrepancies between contig sequences and reference-guided consensus were resolved using Sanger sequencing. Altogether, 20 genomic regions were amplified and Sanger sequenced. Overlapping pools were joined to obtain a complete genome sequence. The sequences of genes containing tandem repeats, i.e. *arp* (*TP_0433*) and *TP_0470* genes, were also resolved using Sanger sequencing. The number of tandem repeats in these genes was estimated using gel electrophoresis. Gene *tprK* (*TP_0897*) showed intra-strain variability and therefore nucleotides in variable regions were replaced with ‘N’s in the complete genome sequence. In addition, the G/C-homopolymeric stretches revealed intra-strain variability throughout the genome. The prevailing number of G/Cs in these regions was used in the final genome sequence. Both protein-coding genes and genes for noncoding RNA were annotated in the genome sequence LMNP-1 based on the annotation of previously published *TPE* strain Gauthier (GenBank: NC_016843.1). Lasergene software was used for strain Gauthier orthologous gene alignment and recalculation of gene coordinates for LMNP-1. A 150-bp gene size limit was applied. Genes were tagged with the *TPELMNP1*-prefix and the locus tag numbering corresponds to the tag numbering of orthologous genes annotated in the *TPE* strain Gauthier genome.

**Supplementary Results**

**Further comparison of whole genome sequences.** All 60-bp repeats in the arp gene of the LMNP-1 genome were of Type II and were identical to other TPE strains previously described. The tprK gene of the LMNP-1 isolate only had three variable regions, V5-V7, when compared to other TPE strains. In addition to differences in TP_0433, TP_0470, and tprK genes, relatively large indels were determined in TPEGAU_0136 (33-nt long deletion; specific for strains Gauthier and Samoa D), in TPFB_0548 (42-nt long deletion; specific for strain Fribourg-Blanc), in TPEGAU_0858 (79-nt long deletion; specific for strain Gauthier), in the intergenic region (IGR) between TPEGAU_0628 and TPEGAU_0629 (302-nt long deletion; specific for strain Gauthier), and in IGR between TPFB_0696 and TPFB_0697 (430-nt long insertion; specific for strain Fribourg-Blanc); the length of other sequence differences ranged between 1-15 nts. Out of a total of 266 strain LMNP nucleotide genome positions different from the sequence of the strain Gauthier (1-302 in length), 28 (10.53%) were located in intergenic regions. The length of intergenic regions in the *TPE* strains (Gauthier, CDC-2, Samoa D, and Fribourg-Blanc) is about 4.63-4.68% (17, 25). In the strain LMNP-1 genome, we detected indel changes in the intergenic regions upstream of genes coding for *Tpr* proteins (*C*, *D*, *G*, *I*, and *J*), chemotaxis proteins (*Mcp* and *CheB*), proteins involved in transport and metabolism (*TmpC*, *SecA*, *EmrE*, and *TP_0925*), ribosomal protein S19 (*RpsS*), RNA polymerase (RpoB), and hypothetical proteins (*TP_0381*, *TP_0383*, *TP_0479*, *TP_0480*, and *TP_0629*). These indel changes were predominantly identified in G/C-homopolymeric tracts and were previously shown to affect transcription rate of the downstream genes (46, 47).

**Supplementary Acknowledgments**

JFG was supported by an NSF Graduate Research Fellowship (DGE-1142336), the Canadian Institutes of Health Research’s Strategic Training Initiative in Health Research’s Systems Biology Training Program, an NSERC Vanier Canada Graduate Scholarship (CGS), and a long-term Research Grant from the German Academic Exchange Service (DAAD-91525837-57048249). HMD was supported by Max Planck Institute for Evolutionary Anthropology and Gent University. JK was supported by the Max Planck Society and the European Research Council (ERC) starting grant APGREID. KIB was supported by a Social Science and Humanities Research Council of Canada postdoctoral fellowship. The PSGS sequencing was supported by the Grant Agency of the Czech Republic to DS and MS (GA17-25455S, GJ17-25589Y). This project was supported by the German Research Foundation through grants FHL (LE1135/2), SK (KN1097/3-1 and KN1097/4-1), and CR (RO3055/2-1). This study was also supported by the AMIDEX project (No. ANR-11-IDEX-0001-02), funded by the 'Investissements d’Avenir' French Government program, managed by the French National Research Agency (ANR) and the Fondation Méditerranée Infection. All raw read files have been deposited in NCBI as part of the BioProject PRJNA343706. Please see supplementary acknowledgments for additional information.

**Supplementary references**

1 Peltzer, A. *et al.* EAGER: efficient ancient genome reconstruction. *Genome Biology* **17**, 1-14, doi:10.1186/s13059-016-0918-z (2016).

2 Li, H. Aligning sequence reads, clone sequences and assembly contigs with BWA-MEM. *arXiv preprint arXiv:1303.3997* (2013).

3 Van der Auwera, G. A. *et al.* From FastQ data to high confidence variant calls: the Genome Analysis Toolkit best practices pipeline. *Curr Protoc Bioinformatics* **43**, 11 10 11-33, doi:10.1002/0471250953.bi1110s43 (2013).

4 Arora, N. *et al.* Origin of modern syphilis and emergence of a pandemic Treponema pallidum cluster. *Nat Microbiol* **2**, 16245, doi:10.1038/nmicrobiol.2016.245 (2016).

5 Tamura, K., Stecher, G., Peterson, D., Filipski, A. & Kumar, S. MEGA6: Molecular Evolutionary Genetics Analysis version 6.0. *Mol Biol Evol* **30**, 2725-2729, doi:10.1093/molbev/mst197 (2013).

6 Kalyaanamoorthy, S., Minh, B. Q., Wong, T. K. F., von Haeseler, A. & Jermiin, L. S. ModelFinder: fast model selection for accurate phylogenetic estimates. *Nat Methods* **14**, 587-589, doi:10.1038/nmeth.4285 (2017).

7 Nguyen, L. T., Schmidt, H. A., von Haeseler, A. & Minh, B. Q. IQ-TREE: a fast and effective stochastic algorithm for estimating maximum-likelihood phylogenies. *Mol Biol Evol* **32**, 268-274, doi:10.1093/molbev/msu300 (2015).

8 Ronquist, F. *et al.* MrBayes 3.2: efficient Bayesian phylogenetic inference and model choice across a large model space. *Syst Biol* **61**, 539-542, doi:10.1093/sysbio/sys029 (2012).

9 Zobanikova, M. *et al.* Whole genome sequence of the Treponema Fribourg-Blanc: unspecified simian isolate is highly similar to the yaws subspecies. *PLoS neglected tropical diseases* **7**, e2172, doi:10.1371/journal.pntd.0002172 (2013).
